# cGMP production of astatine-211-labeled anti-CD45 antibodies for use in allogeneic hematopoietic cell transplantation for treatment of advanced hematopoietic malignancies

**DOI:** 10.1371/journal.pone.0205135

**Published:** 2018-10-18

**Authors:** Yawen Li, Donald K. Hamlin, Ming-Kuan Chyan, Roger Wong, Eric F. Dorman, Robert C. Emery, Douglas R. Woodle, Ronald L. Manger, Margaret Nartea, Aimee L. Kenoyer, Johnnie J. Orozco, Damian J. Green, Oliver W. Press, Rainer Storb, Brenda M. Sandmaier, D. Scott Wilbur

**Affiliations:** 1 Department of Radiation Oncology, University of Washington, Seattle, Washington, United States of America; 2 Clinical Research Division, Fred Hutchinson Cancer Research Center, Seattle, Washington, United States of America; 3 Department of Medicine, University of Washington, Seattle, Washington, United States of America; Los Alamos National Laboratory, UNITED STATES

## Abstract

The objective of this study was to translate reaction conditions and quality control methods used for production of an astatine-211(^211^At)-labeled anti-CD45 monoclonal antibody (MAb) conjugate, ^211^At-BC8-B10, from the laboratory setting to cGMP production. Five separate materials were produced in the preparation of ^211^At-BC8-B10: (1) *p*-isothiocyanato-phenethyl-*closo*-decaborate(2-) (B10-NCS), (2) anti-CD45 MAb, BC8, (3) BC8-B10 MAb conjugate, (4) [^211^At]NaAt, and (5) ^211^At-BC8-B10. The ^211^At-labeling reagent, B10-NCS, was synthesized as previously reported. BC8 was produced, then conjugated with B10-NCS under cGMP conditions to form BC8-B10. [^211^At]NaAt was produced by α-irradiation of Bi targets, followed by isolation of the ^211^At using a “wet chemistry” method. The clinical product, ^211^At-BC8-B10, was prepared by reacting [^211^At]NaAt with BC8-B10 in NH_4_OAc buffer (pH 5.5) for 2 min at room temperature, followed by size-exclusion chromatography purification. Quality control tests conducted on the ^211^At-BC8-B10 included evaluations for purity and identity, as well as pyrogen and sterility tests. Stability of the ^211^At-BC8-B10 in 25 mg/mL sodium ascorbate solution was evaluated at 1, 2, 4, 6 and 21 h post isolation. For qualification, three consecutive ^211^At-BC8-B10 clinical preparations were successfully conducted in the cGMP suite, and an additional cGMP clinical preparation was carried out to validate each step required to deliver ^211^At-BC8-B10 to a patient. These cGMP preparations provided 0.80–1.28 Gbq (21.5–34.5 mCi) of ^211^At-BC8-B10 with radiochemical purity of >97%. The preparations were found to be sterile and have a pyrogen level <0.50 EU/mL. Cell binding was retained by the ^211^At-BC8-B10. ^211^At-BC8-B10 in ascorbic acid solution demonstrated a radiochemical stability of >95% for up to 21 h at room temperature. The experiments conducted have defined conditions for translation of ^211^At-BC8-B10 production from the laboratory to cGMP suite. This study has allowed the initiation of a phase I/II clinical trial using ^211^At-BC8-B10 (NCT03128034).

## Introduction

Allogeneic hematopoietic cell transplantation (HCT) is a widely used form of therapy for patients with advanced hematological malignancies. Although allogeneic HCT can offer the best, and sometimes the only chance for cure, the conditioning regimen often fails to eradicate the target malignancy or is associated with fatal toxicities. It is therefore important to develop new approaches to improve control for diseases such as advanced acute myeloid leukemia (AML), acute lymphoblastic leukemia (ALL), and high-risk myelodysplastic syndrome (MDS) while limiting the toxicity of the conditioning regimen for patients undergoing HCT. One approach that we have investigated is the use of monoclonal antibodies (MAbs) labeled with beta-emitting radionuclides [i.e. iodine-131 (^131^I) or yttrium-90 (^90^Y)] employing radioimmunotherapy (RIT) to deliver high doses of radiation directly to bone marrow (BM), spleen and other affected disease sites, while sparing other organs [1–6]. Although this approach has had success, an attractive alternative is the use of MAbs labeled with alpha-emitting radionuclides for conditioning prior to HCT. RIT with alpha-emitting radionuclides can provide highly localized radiation delivery due to the short path-length of alpha particles, resulting in very high target cell specific cytotoxicity [7–9]. It is our hypothesis that the highly directed cytotoxicity, coupled with a short half-life alpha-emitter, reduces both early toxicity and late complications, such as secondary cancers, associated with conventional systemic conditioning agents and beta-emitter RIT. A particularly attractive alpha-emitting radionuclide for this application is astatine-211 (^211^At). ^211^At has a relatively short half-life (7.21 hours), allowing decay of the radioactivity prior to HCT, and has no alpha-emitting daughter radionuclides that might become free and redistribute to critical organs. Thus, we conducted a number of preclinical investigations into the use of ^211^At-labeled pan hematopoietic anti-CD45 MAbs in HCT [10–12]. Encouraging results from the preclinical studies prompted investigation of the anti-CD45 MAb, BC8, labeled with ^211^At as part of an HCT conditioning regimen for patients with relapsed or refractory leukemia or MDS who have failed conventional front-line therapy.

Herein we report on the methods and materials required for cGMP production of ^211^At-labeled anti-CD45 MAb conjugate, ^211^At-BC8-B10, of interest for clinical trials involving allogeneic hematopoietic transplantation in the treatment of advanced hematological malignancies. Production of ^211^At-BC8-B10 involved five linked production steps, including: (1) production of a boron cage pendant group for conjugation to the anti-CD45 MAb, (2) production of the anti-CD45 MAb under cGMP conditions, (3) production of MAb-B10 conjugate under cGMP conditions, (4) cyclotron production of [^211^At]NaAt, and (5) ^211^At labeling of the MAb-B10 conjugate under cGMP conditions. Information on each production step and quality control measures taken in the cGMP production of ^211^At-labeled BC8-B10 conjugate is provided.

## Materials and methods

### Reagents and general procedures

The chemicals and reagents used were obtained from Sigma Aldrich (St. Louis, MO) or Fisher Scientific (Pittsburgh, PA), and were used without further purification unless otherwise noted. [^125^I]NaI in 0.1 M NaOH was purchased from PerkinElmer (Billerica, MA). Sterile sodium ascorbate (500 mg/mL) was obtained from Milan Institutional (Galway, Ireland). Sterile 0.05 M PBS was obtained from Pharm D Solutions (Houston, TX). Ammonium acetate buffer (0.5 M) was prepared, aseptically vialed and tested under cGMP conditions at the Fred Hutchinson Cancer Research Center Biologics Production Facility. The water used in production of ^211^At-BC8-B10 was purified using a Barnstead EasyPure laboratory water purification system (or equivalent) with resistivity >17.8 MΩ•cm. All chemicals, reagents and other supplies used in the production runs were logged in and physically segregated from other laboratory reagents as required for cGMP materials.

#### ^211^At activity measurement

The quantity and radioisotopic purity of ^211^At was determined using gamma spectroscopy measured on a High-Purity Germanium (HPGe) detector coupled to a PC-based multichannel analyzer (AMETEK, Oak Ridge, TN). The spectra were analyzed using the Maestro-32 software (ORTEC, Oak Ridge, TN). To begin, a HPGe efficiency calibration curve was developed using reference sources Ba-133 (302.8 and 356.0 keV), Cs-137 (661.1 keV), Co-60 (1173.2 and 1332.5 keV) and Na-22 (1274.5 keV) (Eckert & Ziegler, Berlin, Germany). The 687.0 keV gamma ray of ^211^At and the 569.7 and 897.8 keV gamma rays of ^211^Po (T_1/2_ = 0.516 s), a short-lived daughter of ^211^At, were used for quantification of ^211^At radioactivity. The intensities of the primary ^211^At low energy x-ray emissions (76.8 and 79.3 keV) are approximately two orders of magnitude higher than those of the 687.0 (0.261%), 569.7 and 897.8 keV (0.5% and 0.561% from the decay of ^211^Po) gamma rays. Therefore, the reference sealed sources and sample of ^211^At (~150 μCi in 10 μL) was counted at 17 cm above the detector on stainless steel (3 mm thick) and copper (2 mm thick) sheets to attenuate the low energy x-rays. In measurements, at least 10,000 counts for each higher energy gamma peak were taken while keeping the detector dead times ≤ 10%. The 1181.39 keV gamma-ray from ^210^At (I_ɣ_ 99.3%) was used for its quantification, which is evaluated quarterly.

HPGe gamma-ray spectroscopy was used for cross-calibration of three dose calibrators in the production process. After HPGe determination of ^211^At activity, it was decay-corrected to the approximate time (±10 min) of dose calibrator measurement. The calibration setting number was adjusted until the screen displayed the expected activity. The calibrator numbers for ^211^At for Capintec CRC-15R, CRC-55tR and CRC-12R were determined to be 42, 44 and 48, respectively. Cross-calibrations are conducted quarterly, and it should be noted that small differences (< 5%) in the calibration setting have been observed. Also, calibration was conducted on activity in the size/type of syringe to be used for administration of the ^211^At-BC8-B10 to patients.

#### Production of B10-NCS

The bifunctional conjugate used for labeling MAbs with ^211^At, *p*-isothiocyanato-phenethyl-*closo*-decaborate(2-) (designated as B10-NCS) was prepared in a four step procedure (S1 Fig), as previously described [13]. B10-NCS was used as a raw material for producing cGMP grade BC8-B10. Each batch was prepared within a day of its use in the cGMP conjugation. Freshly purchased reagents (previously unopened bottles) were used for the syntheses of B10-NCS. Confirmation of identity and purity of the B10-NCS was required for release to the BC8-B10 production step. Identity and purity were assessed by reversed-phase high performance liquid chromatography (RP-HPLC) (S2 Fig) using previously prepared reference standards. High resolution negative ion mass spectral analysis was also performed to confirm identity of the B10-NCS (S3 Fig). These data were collected for each batch of B10-NCS used to prepare cGMP grade batches of B10-BC8. A Certificate of Analysis was provided with the B10-NCS, attesting that the compound had the correct identity, mass and purity.

#### Production of BC8 and BC8-B10

BC8 MAb and BC8-B10 MAb conjugate were prepared under cGMP conditions. Clinical grade murine anti-CD45 monoclonal antibody BC8 was produced in high purity (S4 Fig) as previously described [2]. The bulk purified BC8 was sterile filtered and stored at 4 ºC prior to conjugation. Conjugations of B10-NCS to BC8 were conducted as previously described for conjugation to an anti-canine CD45 MAb [11]. Briefly, from 9 mg to 2.37 g of purified BC8 and 5 molar equivalents of the B10-NCS reagent were added to a solution of HEPES buffer (100 mM pH 8.5). The reaction was run overnight at room temperature with gentle tumbling. The BC8-B10 was purified on a size exclusion column using PBS as eluent. For small quantities of BC8-B10, PD-10 columns (GE Healthcare, Marlborough, MA) were used. The eluted BC8-B10 fractions were pooled. For larger quantities of BC8-B10, the samples were concentrated utilizing a 30 kDa Kvickstart Cassette (GE Healthcare) to buffer exchange and concentrate BC8 into 100mM HEPES pH 8.5, then subsequent ultrafiltration and diafiltration of BC8-B10 into phosphate buffered saline (PBS). Clinical grade BC8-B10 utilized a Spectrum Hollow Fiber Filter (Spectrum, Inc., Rancho Domingues, CA) for purification. Purity of BC8-B10 was assessed using size-exclusion HPLC (S5 Fig). Retention of cell binding after conjugation was assessed using flow cytometry on a Guava EasyCyte Mini (Millipore Sigma, Burlington, MA) (S6 Fig). IEF analyses were conducted on a XCell SureLock Mini-Cell Electrophoresis system using pH 3–10 IEF gels (Thermo Fisher Scientific, Waltham, MA) to determine how isoelectric point (pI) of BC8 changed after conjugation of B10-NCS (S7 Fig). The extent of BC8 modification during conjugation with B10-NCS was estimated from IEF. Densitometric analyses on the IEF bands relative to unmodified IEF bands indicated that less than 3% remained as unconjugated BC8 (S8 Fig). Mass spectral analyses were conducted on an Applied Biosciences SciEx 4800 Matrix Assisted Laser Desorption/Ionization Time of Flight (Maldi TOF/TOF) Analyzer (Wellborn TX) to provide a better estimate of the number of B10 conjugates per BC8 molecule. That estimation was conducted by subtracting an average mass of two BC8 MS runs from an average mass of two BC8-B10 MS runs, then dividing that difference by the mass of a B10-NCS moiety (S9 Fig). After production, clinical grade BC8-B10 dispensed into 5 mL sterile vials at a concentration of 5.0 mg/mL in phosphate buffered saline (PBS). The vialed BC8-B10 was examined by SE-HPLC, SDS-PAGE and IEF for identity; tested for sterility, endotoxin and general safety; and functionality was examined by radiolabeled cell binding assay, before being released for clinical preparations.

#### Production of ^211^At

^211^At was produced by irradiation of Bi metal on an aluminum target support with 29 MeV α-particles using the Scanditronix MC50 cyclotron as previously described [14]. After irradiation, ^211^At was isolated from the Bi target using a “wet chemistry” approach [15] in a single use glovebox (Innovative Technologies, Inc) vented through a charcoal filter on the glovebox exhaust and through a second charcoal-filtered Plexiglas enclosure (Biodex 112–038) within a radiochemical fume hood. In brief, the wet chemistry isolation procedure involved the following steps: (1) the bismuth target was completely dissolved in concentrated HNO_3_, (2) the HNO_3_ solution was evaporated to dryness at 300°C, leaving a white residue, (3) the residue was re-dissolved in 8 M HCl, (4) diisopropyl ether (DIPE) was used to extract ^211^At from the aqueous phase, (5) ^211^At was back-extracted from DIPE into 4 M NaOH, (6) the pH of the aqueous NaOH solution was adjusting to ~ 7.0 using aliquots of 0.1, 0.5, 1 or 4 M HCl, and (7) finally, the neutralized ^211^At solution was reconstituted to 0.05 M NaOH using 1 M NaOH.

Assessment of the radiochemical purity of isolated ^211^At was accomplished by ion-exchange (IE) radio-HPLC (S10 Fig) and iTLC analyses (S11 Fig). Analyses were performed on ^211^At solutions and on ^125^I standards ([^125^I]iodide and [^125^I]iodate) for peak retention time comparison. HPGe measurements were performed on isolated ^211^At solutions to verify that no ^210^At was co-produced.

#### Production of ^211^At-BC8-B10

During the cGMP production of ^211^At-BC8-B10 all radiolabeling experiments and purification steps were conducted in a fume hood for safety considerations. Production of ^211^At-BC8-B10 was conducted under aseptic conditions. ^211^At-labeling of BC8-B10 was conducted by sequential addition of 1.4 mL of 0.5 M ammonium acetate (pH 5.5) and 1.6 mL of 5 mg/mL BC8-B10 solution (8 mg) to an ^211^At / 0.05 M NaOH solution (~ 0.7 mL) in a sterile glass vial. The reaction was run at room temperature with gentle shaking for 2 min using a timer. Then 200 μL of 1 mg/mL sodium thiosulfate was added to the reaction vial, followed immediately by 200 μL of 500 mg/mL ascorbate solution. The ^211^At-BC8-B10 was purified using three Sephadex PD-10 columns, eluting each column with 4 mL PBS/sodium ascorbate. The ^211^At-BC8-B10 was eluted into a sterile vial, followed by withdrawal of that solution into a sterile syringe through a 0.1 μm filter (Millex-VV, Millipore Sigma, St. Louis, MO) for administration. Small aliquots were taken from the elution vial to assess purity by iTLC and obtain protein quantification by HPLC. Additional samples were taken after filtration for endotoxin, sterility and immunoreactivity assessments, as described below.

Protein Concentration. Radio-SE-HPLC was used to determine the protein concentration of the ^211^At-BC8-B10. This determination was accomplished based on assessment of the area under the protein peak (UV, 280 nm) relative to a standard curve prepared from three samples with known concentrations of protein measured on the same day. The HPLC system consisted of a Hewlett-Packard Variable Wavelength Detector (280 nm), a Beckman Model 170 Radioisotope Detector, isocratic pump, and a Protein-Pak glass 300SW column (7.5mm x 300 mm, 10 μm; Waters Corp., Milford, MA). The column was eluted using 1x Dulbecco’s phosphate buffered saline at a flow rate of 1 mL/min. The retention time for the ^211^At-BC8-B10 is 8.2–8.6 min using this system.

^211^At-BC8-B10 purity. The radiochemical purity of the product was determined using iTLC analyses. This test used iTLC-SG chromatographic strips (Agilent, Santa Clara, CA) eluting with normal saline (0.9% w/v of NaCl). Protein-associated activity remained at the origin and free ^211^At moved with the solvent front. After elution, the iTLC strips were cut into three pieces and counted separately on an automated Wallac Wizard gamma counter (PerkinElmer, Waltham, MA). The radiochemical purity was determined by the percentage of counts in the product peak (origin) compared to the total counts of the three iTLC strip pieces.

Immunoreactivity Assessment. Immunoreactivity was assessed using a radiolabeled cell binding assay and competitive binding was evaluated by flow cytometry. The buffer used in the radiolabeled cell binding assay was RPMI 1640 cell culture medium with 0.1% NaN_3_. The radiolabeled cell binding assay involved Ramos cells (CD45^+^ lymphoma cell line) plated at a concentration of 30 x 10^6^/well in a 96-well V-plate on ice and incubated at 4°C for a minimum of 30 min. All tubes, solutions and cells were otherwise kept ice-cold throughout the experiment. The cells were centrifuged at 1000 rpm and the supernatant was discarded. BC8-B10 and binding buffer were added to some wells, then the plate was incubated on ice for 45 min. Following that, ^211^At-BC8-B10, ^125^I-BC8-B10 and an ^125^I-labeled isotype-matched non-binding control MAb, ^125^I-BHV1, radioiodinated as previously described [13], were added to the wells and the plate was incubated on ice for 1 h with gentle mixing/vortexing every 15 min. After incubation, the cells were centrifuged at 1000 rpm and the supernatant was discarded. The cells were washed two times using the binding buffer, then cell-associated radioactivity was measured by counting the cell pellets on a Wizard Gamma Counter.

Flow cytometry was conducted on Ramos cells (1 x 10^6^/well) incubated with 5 μg/mL of BHV1, BC8-B10, ^125^I-BC8-B10 or ^211^At-BC8-B10 at 4°C for 30 min in PBS / 2% Fetal Bovine Serum (PBS/FBS). The cells were washed twice with PBS / FBS and incubated at 4°C for 30 min using 4 μL of secondary R-Phycoerythrin Goat anti-Mouse IgG antibody. After 2 more washes, samples were analyzed using a Guava EasyCyte Mini Flow Cytometer (Millipore Sigma, Burlington, MA).

Pyrogenicity Testing. Testing of pyrogen levels in ^211^At-BC8-B10 was performed using the Limulus Amebocyte Lysate (LAL) endotoxin test in an Endosafe-PTS instrument (Charles River Laboratory, Wilmington, MA). The test was conducted per manufacturer’s instructions on FDA-licensed cartridges held at 37°C for 5 min, followed by automated processing and spectrophotometric readout. The product passes the pyrogen test if the endotoxins present is under the limit of detection (<0.5 EU/mL) or have ≤ 5 EU/kg/h at product infusion.

Sterility Testing. Sterility testing was conducted in the University of Washington Medical Center Clinical Microbiology Laboratory. The tests were run on 2 mL of 0.1 μm-filtered ^211^At-BC8-B10 solution. The sterility test was conducted by inoculation of tubes containing Fluid Thioglycolate Medium and Soybean-Casein Digest Medium (TSB) as recommended in the US Pharmacopeia. Thioglycolate tubes were incubated at 37°C and TSB tubes were incubated at room temperature for 14 days. The samples were assessed at days 3, 7 and 14 post production.

Filter integrity Testing. Since sterility testing was not possible before administration, a post-use filter integrity test (bubble point test) was conducted after filtering the ^211^At-BC8-B10 to assure that the 0.1 μm filter remained intact. The filter integrity test was accomplished by connecting the 0.1 μm sterile filter used in filtering the final ^211^At-BC8-B10 product to a compressed air cylinder via two regulators in tandem (S12 Fig). Tubing downstream of the filter was immersed into water and compressed air pressure was gradually increased by adjusting the second regulator until bubbles were observed. The filter was considered intact if the bubble point was ≥ 62 psi.

## Results

**Fig 1:**
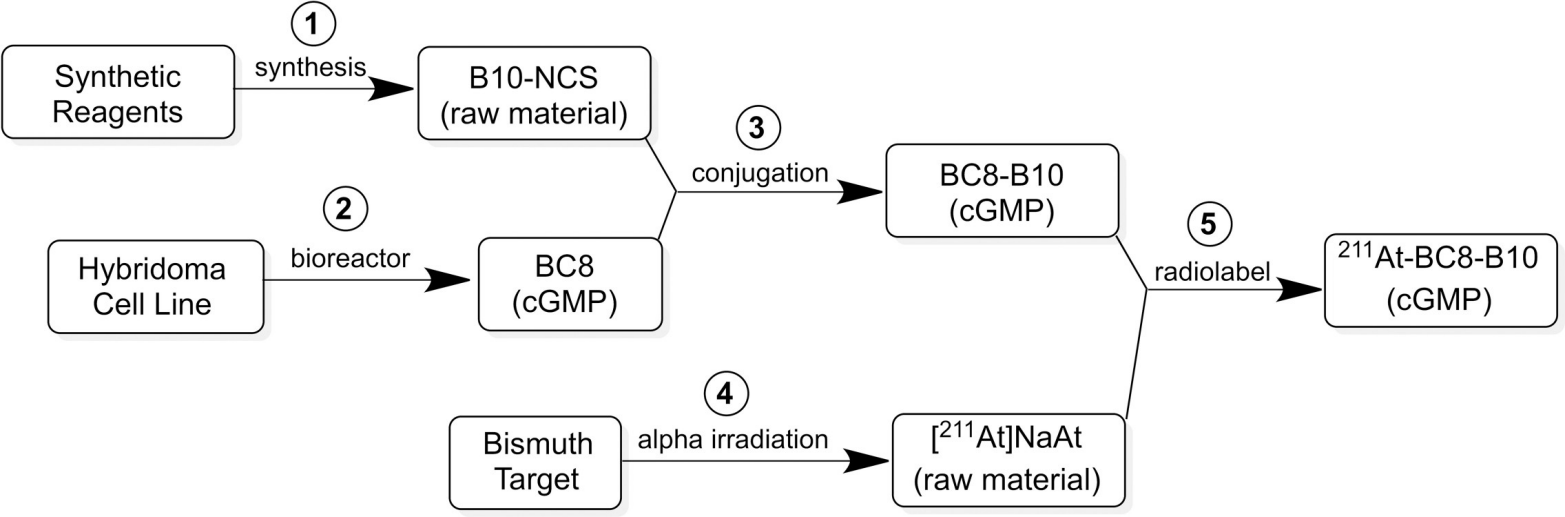
Scheme depicting the five production steps in the cGMP production of ^211^At-BC8-B10. Each of the products were isolated, purified and characterized. The product from each step had to meet preset release criteria prior to being used in the next step.

None of the components required for production of ^211^At-BC8-B10 were available from commercial sources, so five different production steps were required to generate the final radiopharmaceutical product, as outlined in Fig 1. In each production step the product was isolated, purified and analyzed to meet our preset release criteria for identity and purity prior to being used in the next step. The following text describes methods used in each step to produce the four intermediate products and the clinical grade ^211^At-BC8-B10.

### Production of B10-NCS (step 1)

The B10-NCS reagent used to conjugate with MAbs for ^211^At-labeling was prepared in four synthetic steps as previously described [13]. Concerns about the stability of an isothiocyanate functionality in the presence of the dianionic decaborate cage led to a decision that the aniline intermediate would be stored rather than the isothiocyanate-containing B10-NCS. Thus, a quantity (i.e. 1.2 g) of the intermediate aniline derivative was prepared, purified and vialed in smaller quantities (5–50 mg) for storage at 4°C. Conversion of the aniline to an isothiocyanate functionality was done just prior to use of the B10-NCS in conjugation reactions. HPLC chromatography was used to verify the identity of the B10-NCS and show that its purity was greater than 90% (generally >98%). Additionally, negative ion electrospray mass spectra (ESI-MS) analysis confirmed the identity of the isolated molecule. The B10-NCS met release criteria and was provided, with a Certificate of Analysis, for preparation of cGMP grade BC8-B10.

### Production of BC8 and BC8-B10 (steps 2 and 3)

The anti-CD45 MAb BC8 was produced in the Biologics Production Facility (FHCRC, Seattle) in high purity under cGMP conditions, as previously described [2]. The next step, conjugation of BC8 with B10-NCS, represents the first time this critical translational step has been successfully performed under cGMP conditions. BC8 conjugation studies conducted under general laboratory conditions had shown that 10 equivalents of B10-NCS yielded conjugates which retained immunoreactivity. However, when a pilot study using 10 equivalents of B10-NCS for cGMP production of BC8-B10 was conducted, a significant decrease in cell binding was noted by flow cytometric analysis. IEF analysis of BC8-B10 showed that the pilot cGMP production had loaded more B10 moieties per BC8 molecule than obtained in the previous laboratory conjugations (i.e. lower pI obtained). Evaluation of reactions of BC8 with 5, 10 and 20 equivalents of B10-NCS by IEF indicated that differing amounts of B10-NCS had been conjugated, as expected. Mass spectral data provided estimates of 2.6, 5.6 and 7.3 B10-NCS moieties conjugated (respectively). The BC8-B10 produced by reaction with five equivalents of B10-NCS appeared most favorable, and ^211^At-labeling of this BC8-B10 product provided >80% radiochemical yield while retaining immunoreactivity. Based on those results, a pilot batch of BC8-B10 was produced by reaction of BC8 with five equivalents of B10-NCS at room temperature for 16 hours. The resulting BC8-B10 was purified using size exclusion chromatography. Identity, purity and immunoreactivity tests passed release criteria, and a full cGMP production conjugation of BC8 was conducted. SE-HPLC, IEF and mass spectral analyses were conducted on that batch. SE-HPLC analyses indicated the monomeric contents of both BC8 and BC8-B10 were >90%. IEF analyses confirmed that virtually all of the BC8 had been converted to BC8-B10. Mass spectral analyses of BC8-B10 samples from two clinical batches indicated that on average 2.7 B10-NCS moieties (each) were conjugated per MAb molecule. FACS analysis of the BC8 and BC8-B10 indicated that >70% of the immunoreactivity was retained. Subsequent production runs of BC8-B10 used up to 2.37g of BC8. All batches of BC8-B10 met release criteria.

### Production of ^211^At (step 4)

Production of ^211^At was accomplished by alpha-irradiation of bismuth targets at 29 MeV. In five clinical preparations integrated currents of 130 to 180 μAh, obtained over a 3.6 to 5 h time period, produced 2.49 GBq (67.3 mCi) to 4.37 GBq (118 mCi) of ^211^At at end of bombardment. These quantities were estimated from dose calibrator readings x 1.3 to compensate for bismuth attenuation [15]. After conducting the wet chemistry isolation procedure [15] and quality assurance procedures, 1.14 GBq (30.9 mCi) to 2.54 GBq (68.7 mCi) of [^211^At]NaAt in 0.05 M NaOH solution was delivered to the cGMP suite for astatination of BC8-B10. In each ^211^At production run, samples of the isolated ^211^At were analyzed by radio-HPLC and/or radio-iTLC to verify that the percent [^211^At]astatide was greater than 85%. Also, a sample of isolated ^211^At is evaluated on the HPGe detector quarterly to assess the isotopic purity of the product. To date, all analyses have shown the radionuclidic purity of ^211^At to be > 99.99%, with no detectable ^210^At observed in the HPGe measurements.

### Production of ^211^At-BC8-B10 (step 5)

An initial ^211^At-labeling experiment was conducted to assess ^211^At-BC8-B10 scale-up and purification methods. In a non-GMP scale-up study of BC8-B10, 2.13 GBq (57.4 mCi) of ^211^At was obtained using the standard “wet chemistry” isolation method. ^211^At labeling of BC8-B10 was conducted for 1 minute at neutral pH without added oxidant, followed by quenching using thiosulfate. After purification by size-exclusion chromatography (PD-10), 1.12 GBq (30.3 mCi) of ^211^At-BC8-B10 was obtained (53% radiochemical yield). In the purification step, ^211^At-BC8-B10 was eluted from a PD-10 column using a solution of PBS and ascorbic acid (~25 mg/mL) to minimize radiolysis of the labeled antibody. Ascorbate has a strong UV absorbance at 280 nm and complicates quantification of the amount of protein in the final product. Therefore, a method was developed using UV absorbance of the labeled BC8-B10 obtained from a SE-HPLC chromatogram. In the analysis, the area of the UV absorbing peak on the chromatogram was compared with a standard curve from known quantities of BC8-B10 measured on the same day (S13 Fig). The ^211^At-BC8-B10 obtained was a single peak (no higher molecular weight species) by radio-SE-HPLC, validating that the process was ready for ^211^At labeling in the cGMP suite.

**Fig 2:**
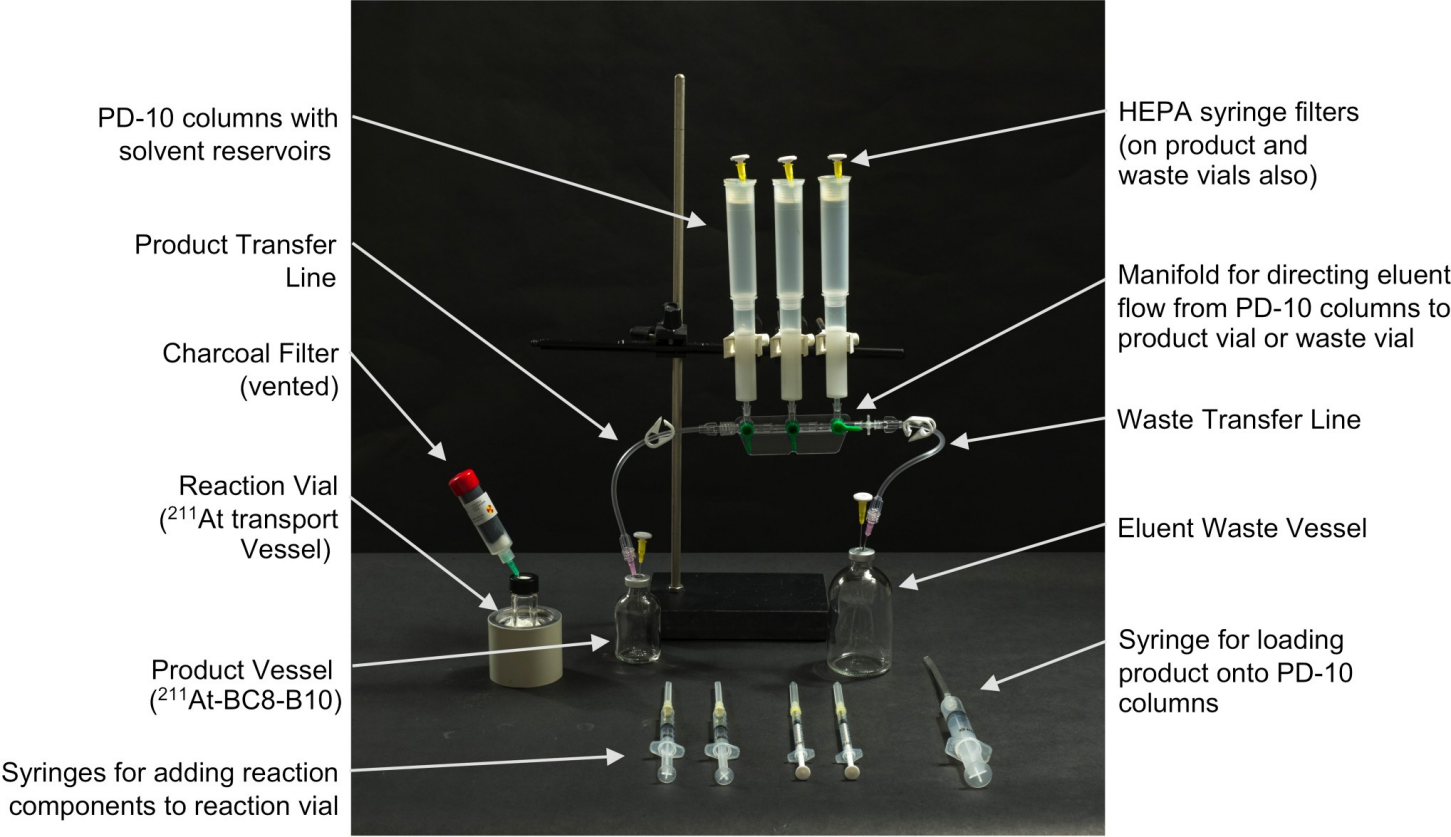
Picture showing setup and transfer syringes used for production and purification of ^211^At-BC8-B10. Reagent vials containing (1) ammonium acetate, (2) BC8-B10, (3) sodium thiosulfate, and (4) sodium ascorbate are not shown. Those reagents were added via syringe to the reaction vial containing ^211^At. Once the additions were made, the reaction solution was taken up into the large syringe and 1/3 volume was added to the top of each column. Following that the pre-rinsed columns were sequentially eluted with 4 mL of sterile PBS/sodium ascorbate solution. Fractions containing the ^211^At-BC8-B10 were collected in the product vessel and other elution volumes were directed to the waste vessel.

As part of the initial setting up of the cGMP suite, experiments were conducted to develop a method for the purification of ^211^At-BC8-B10 using a sealed apparatus containing PD-10 size exclusion columns. Due to the volume of the reaction mixture in the radiolabeling procedure, ~4 mL, three PD-10 columns were required in the purification process. After some testing of different designs, it was determined that transferring the labeled antibody from the reaction vial to the PD-10 columns was best conducted using a sterile syringe with a long needle to place ~1/3 of the reaction solution directly onto the frit in each PD-10 column. Elution of the three columns into a single product vial was accomplished using a manifold with three stopcocks, as depicted in Fig 2. Once the purification setup had been tested, a successful initial trial production run was carried out in the cGMP suite. That trial production run was followed by three consecutive qualification ^211^At-BC8-B10 production runs conducted under the same conditions. All of the qualification ^211^At-labeling runs were successful. An additional trial clinical preparation was performed to run through production of a clinical dose of ^211^At-BC8-B10 starting from a patient order through delivery of the filled syringe to the administration room. The results of those five runs are summarized in Table 1. In the production runs, up to 4.37 GBq (118 mCi) of ^211^At and 1.28 GBq (34.6 mCi) of ^211^At-BC8-B10 were prepared. The average ^211^At percent yield of ^211^At isolation was 52 ± 9% (non-decay corrected) and the average percent isolated yield of ^211^At-BC8-B10 was 61±16%.

**Table 1:** Quantities in GBq (mCi) and yields for preparation of [^211^At]NaAt and ^211^At-BC8-B10.

Run # (Trial or Qualification)	^211^At Produced GBq (mCi)*	Isolated Yield of ^211^At**	^211^At delivered to cGMP room GBq (mCi)	^211^At-BC8-B10 Produced GBq (mCi)	Radiochemical yield**
1 (T)	4.19 (113.0)	67%	2.54 (68.7)	0.80 (21.5)	38%
2 (Q)	2.49 (67.3)	51%	1.14 (30.9)	0.71 (19.1)	67%
3 (Q)	3.12 (84.2)	56%	1.49 (40.2)	0.66 (17.9)	47%
4 (Q)	3.93 (106)	43%	1.51 (40.8)	1.17 (31.5)	80%
5 (T)	4.37 (118)	44%	1.75 (47.2)	1.28 (34.5)	73%

*Dose calibrator reading x 1.3 to correct for bismuth metal attenuation (see ref. #^15^)

**Actual amount isolated–not decay corrected

**Fig 3:**
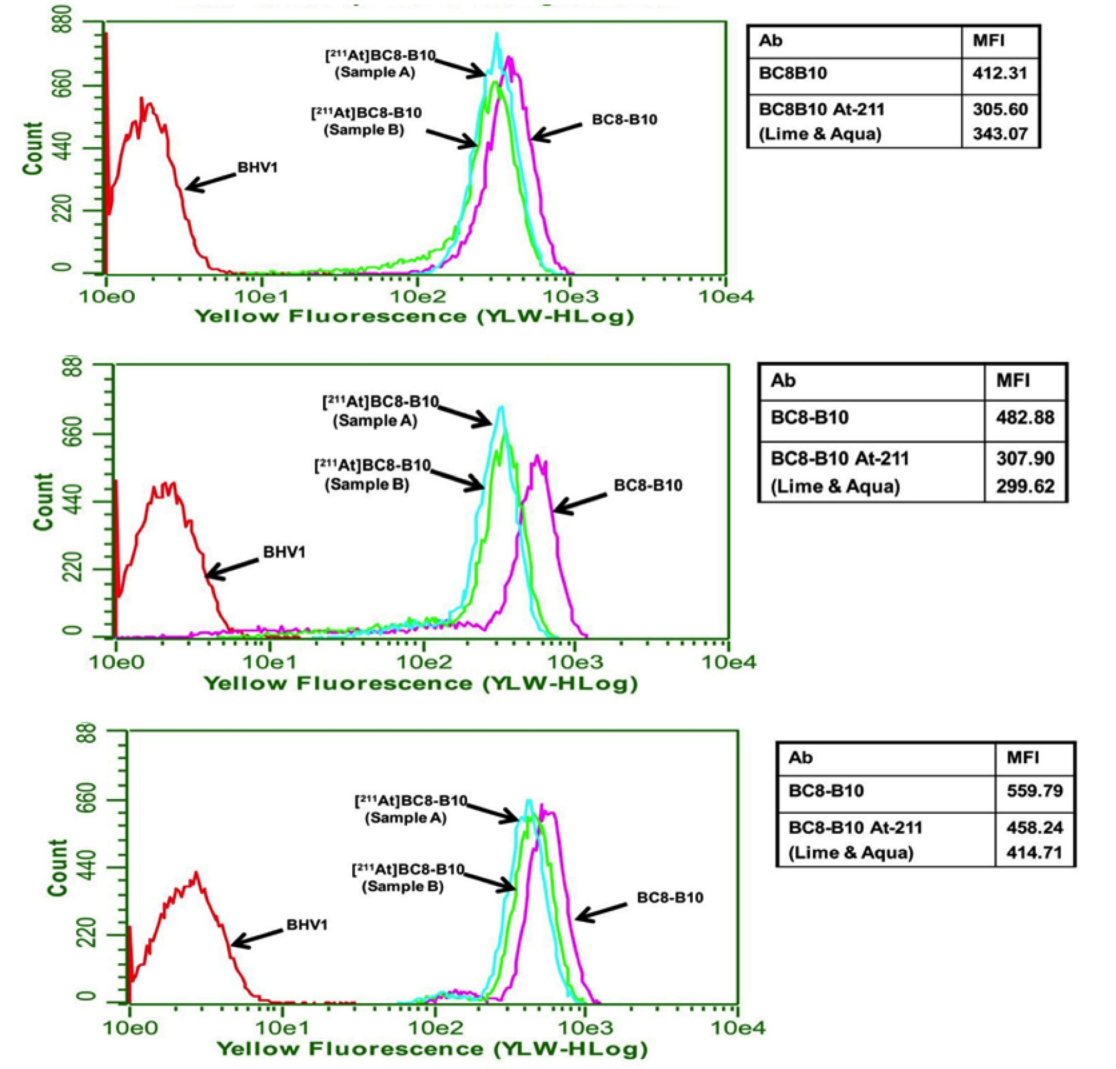
FACS histograms of ^211^At-BC8-B10, BC8-B10 and BHV1 (isotype matched control) binding to Ramos cells. Duplicate samples of ^211^At-BC8-B10 were obtained from three production runs used to qualify the radiolabeling process. The ratio of mean fluorescence intensity (MFI) values for the ^211^At-BC8-B10 to BC8-B10 provides information on how the antibody binding has been affected by the radiolabeling process. A value of >50% MFI by flow cytometry for CD45 positive cell binding of the ^211^At-BC8-B10 relative to non-labeled BC8-B10 is used as the cutoff for release using this assay. This value has been used previously in clinical trials with ^131^I-labeled BC8.

Results from radiochemical purity, pyrogen and sterility tests for the three qualification production runs are shown in Table 2. After PD-10 purification, the radiochemical purity in all ^211^At-BC8-B10 preparations was higher than 97%. The endotoxin levels in the prepared doses were below the limit of detection. All ^211^At-BC8-B10 preparations were found to be sterile at the 3-, 7- and 14-day post isolation test times. Cell binding analyses of ^211^At-BC8-B10 from the three qualification preparations relative to BC8-B10 were 79%, 63% and 78%, respectively, as shown in Fig 3.

**Table 2:** Purity, pyrogenicity and sterility results from the ^211^At-BC8-B10 qualification runs.

Sample ID	Radiochemical Purity by iTLC (time = 0 hours)	Pyrogen Test results (Endosafe-PTS)	Sterility (UW Microbiology Lab) TSB/Thio*		
			Day 3	Day 7	Day 14
Qualification run 1	98.4%	< 0.500 EU/mL	Neg/Neg	Neg/Neg	Neg/Neg
Qualification run 2	97.6%	< 0.500 EU/mL	Neg/Neg	Neg/Neg	Neg/Neg
Qualification run 3	98.1%	< 0.500 EU/mL	Neg/Neg	Neg/Neg	Neg/Neg

*Sterility using Tryptic Soy Broth (“TSB”) or Thioglycolate Media (“Thio”)

### Stability of ^211^At-BC8-B10

**Fig 4:**
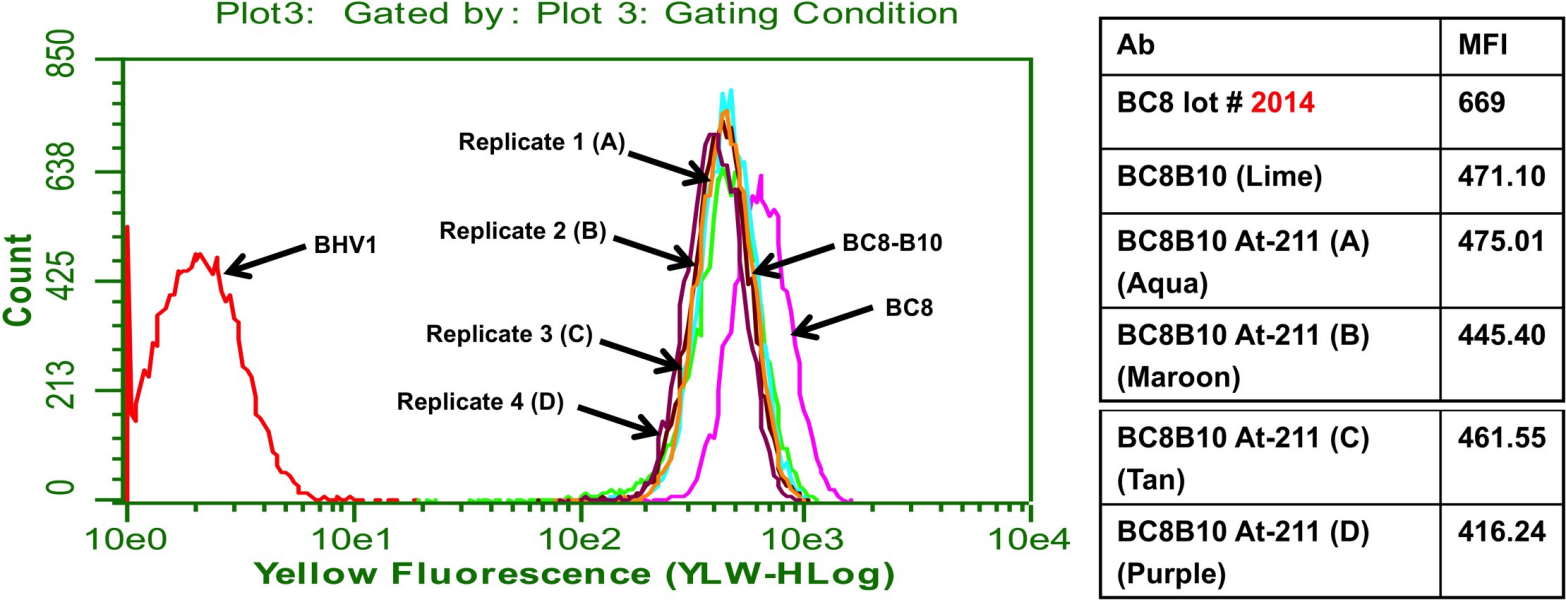
FACS binding histograms for four different ^211^At-labeled BC8-B10 preparations (containing ascorbic acid) after incubating 4 h at room temperature.

**Fig 5:**
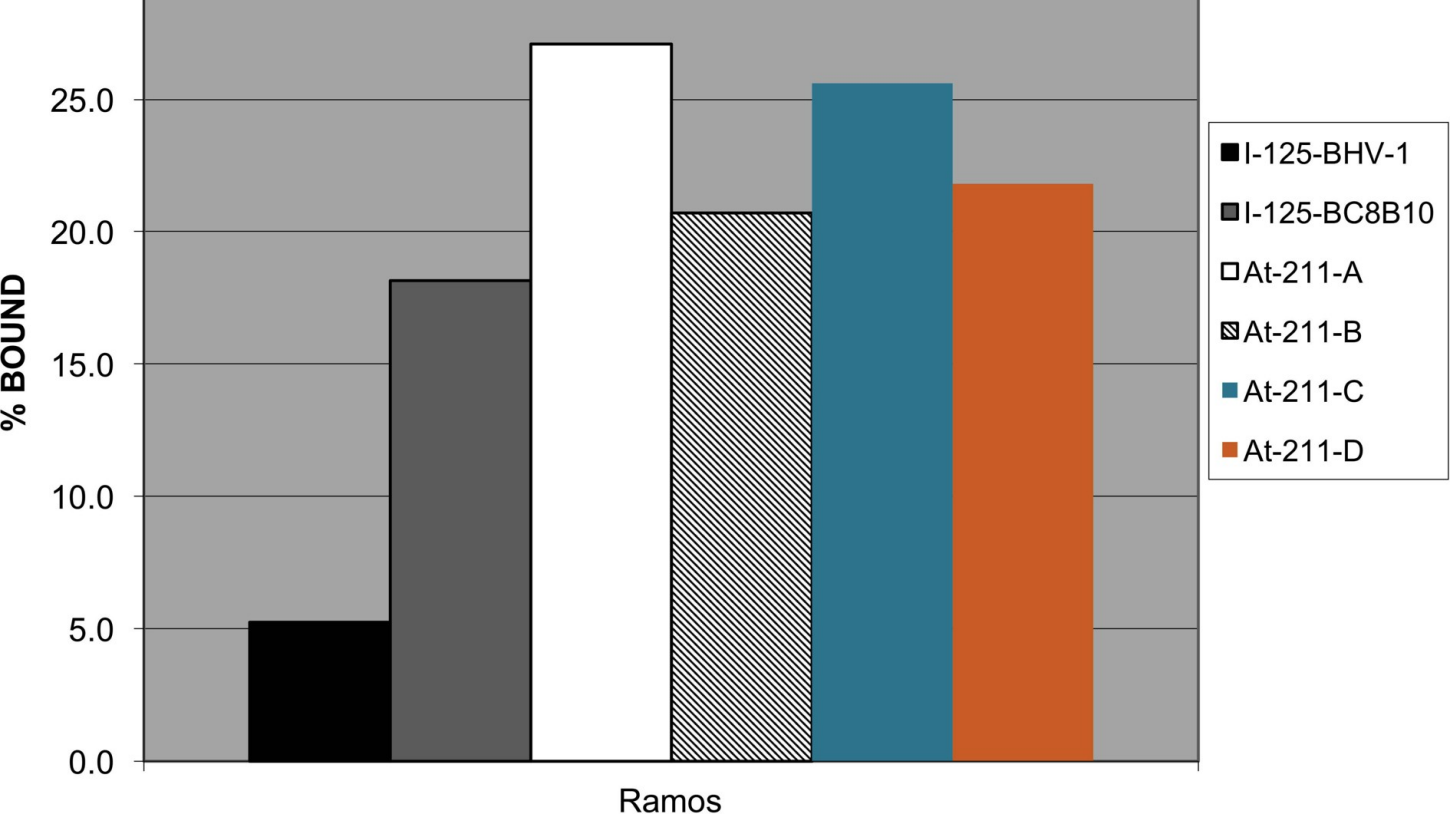
Bar graph showing Ramos cell binding for ^211^At-BC8-B10 after being kept at room temperature for 4 h. ^125^I-labeled BC8-B10 was used as a reference standard and ^125^I-labeled BHV-1 was used as an isotype-matched non-binding control.

Four preparations of ^211^At-BC8-B10 were conducted under non-cGMP conditions to evaluate stability in sodium ascorbate solution (~25 mg/mL) at 1, 2, 4, 6 and 21 hours post isolation. The results shown in Table 3 indicate the radiochemical purity of the ^211^At-BC8-B10 remains above 95% after being maintained at room temperature for 21 hours. Since our clinical preparations will be administered within 4 hours, immunoreactivity testing was limited to that time period of time. Flow cytometry and radiolabeled cell binding confirmed that after 4 hours the ^211^At-BC8-B10 in sodium ascorbate solution retained cell binding affinity. ^211^At-BC8-B10 cell binding assessed by flow cytometry was not distinguishable from unlabeled BC8-B10 (Fig 4). Fig 5 shows that CD45 positive Ramos cell binding of ^211^At-BC8-B10 is similar to that of a control ^125^I-labeled BC8-B10, and significantly higher than that of ^125^I-labeled BHV-1 (negative isotype-matched control MAb).

**Table 3:** Results from ^211^At-labeled BC8-B10 stability tests.

Sample ID	Specific Activity MBq/mg (mCi/mg)	Radiochemical Purity by iTLC				
		1 h	2 h	4 h	6 h	21 h
Prep A	59.2 (1.60)	98.5%	97.7%	97.4%	97.2%	96.0%
Prep B	62.9 (1.70)	98.4%	97.9%	97.4%	97.3%	96.8%
Prep C	62.9 (1.70)	98.3%	97.5%	97.0%	96.7%	96.7%
Prep D	159 (4.29)	97.8%	97.5%	96.7%	97.6%	95.7%

## Discussion

^211^At is one of only a few alpha-particle emitting radionuclides with radiochemical properties suitable for use in treatment of human disease [16]. While a large number of preclinical studies have been conducted to evaluate the use of ^211^At in the treatment of cancer, to date there have been few examples of translation of the preclinical findings to clinical studies. The first documented use of ^211^At treatment in humans was conducted in 1954 to evaluate its’ potential in treatment of thyroid disorders [17]. Another example of the therapeutic use of ^211^At was reported in 1990, wherein a single patient was treated intra-arterially with ^211^At-labeled human serum albumin microspheres as a palliative measure for inoperable lingual carcinoma [18]. More recently, two clinical trials have been conducted to evaluate the use of ^211^At-labeled MAbs in the treatment of patients with non-hematologic malignancies. In a clinical trial conducted at Duke University, eighteen patients with recurrent malignant brain tumors had the ^211^At-labeled anti-tenascin MAb 81C6 administered locally into the surgically created resection cavities [19]. The other reported clinical trial was conducted in Sweden at the University of Gothenburg. In that trial, ^211^At-labeled MX35 F(ab´)_2_, targeting the sodium-dependent phosphate transport protein 2b, was administered via peritoneal catheter to nine patients with ovarian cancer [20]. The studies described herein to produce ^211^At-BC8-B10 are significantly different from the previously prepared ^211^At-labeled MAbs.

While the two other clinical trial preparations of ^211^At-labeled MAbs described above have used dry distillation to isolate ^211^At from irradiated bismuth targets, we experienced difficulties in reproducibility using that approach for isolation of ^211^At. Therefore, a “wet chemistry” approach for isolation of ^211^At from irradiated targets was developed [15]. Isolation of ^211^At from irradiated Bi targets using the “wet chemistry” method has provided consistently high isolation yields for over 200 ^211^At production runs in our laboratory during the past 10 years. Additionally, an instability of astatinated benzoate MAb conjugates observed in preclinical studies led to development of a boron cage pendant group for ^211^At-labeling of MAbs [13, 21–23]. In those studies, it was found that conjugation of a bifunctional molecule, referred to as B10-NCS, provides very high in vivo stability in ^211^At-labeled MAb-B10 conjugates [13]. Further, the use of B10-NCS conjugates can provide higher overall radiolabeling yields than obtained when the stannylbenzoate moieties are used for radiolabeling. While many preclinical studies were conducted using the wet chemistry isolation approach and B10-NCS MAb conjugates, these procedures had not been used previously in clinical preparations.

The primary goal of this investigation was to translate our general laboratory labeling approach to cGMP production of an ^211^At-labeled anti-CD45 MAb conjugate, ^211^At-BC8-B10, for use in a clinical trial. The translation required a number of procedural changes from those used in preclinical ^211^At-labeling experiments. For example, in preclinical labeling experiments the ^211^At solution was neutralized; however, it was found that a basic solution of ^211^At (in 0.05 M NaOH) was required for transfer of ^211^At solution to the clinical labeling suite because ^211^At recovery from a neutral solution was low otherwise. The low recovery came about from ^211^At adhering to or reacting with the wall of the vial during transportation and experimental setup in the cGMP facility. However, when the ^211^At solution was basic (e.g. 0.05 M NaOH), full recovery of the activity was possible. Prior to radiolabeling, sterile NH_4_OAc buffer (0.5 M, pH 5.5) was used to adjust the ^211^At solution to a neutral or slightly acidic solution for radiolabeling. Another example of a procedure change involved changing the order of reagent addition from that used in the preclinical labeling experiments. In the clinical preparation of reagents, ammonium acetate buffer and antibody, were added sequentially to ^211^At to minimize loss of ^211^At. A third example of a change made was the use of septum-piercing sterile syringes and sterile glass vials with septum caps rather than pipets and plastic vials, as the later are incompatible with the sealed PD-10 column setup used. Yet another very important modification was the addition of an antioxidant to the column elution mixture and final solution of ^211^At-BC8-B10 to minimize formation of protein dimers or aggregates. Ascorbic acid in high concentration (25 mg/mL) was used as an effective intervention to ameliorate this problem [24].

Ultimately, we successfully developed a procedure for preparing ^211^At-labeled BC8-B10 using aseptic techniques under cGMP conditions. In the investigation five ^211^At-BC8-B10 clinical grade production runs were conducted under cGMP conditions. Two of the production runs were conducted to assess the steps in the labeling process, and were deemed trial runs. The other three cGMP ^211^At-BC8-B10 production runs were high-level ^211^At qualifications production runs required for filing an IND application. We were able to obtain good radiolabeling yields and high radiochemical purities using the procedure, and the products involved in the overall production process passed all requisite QA/QC tests for release. S1 Table in Supporting Information provides a list of release criteria used in each step. The time required for obtaining cell binding results by FACS or radiolabeled cell binding (2–3 h) is long compared with the 7.21 hour half-life of ^211^At. Therefore, the immunoreactivity of ^211^At-BC8-B10 is not feasible for use as a release criterion for patient administration. Cell binding analyses are nevertheless performed to assess the immunoreactivity of ^211^At-BC8-B10 in each preparation, and the results are reviewed retrospectively to inform future ^211^At labelings.

## Conclusions

Our objective in this investigation was to demonstrate production of ^211^At-BC8-B10 under cGMP conditions, such that a clinical trial using that radiopharmaceutical for treatment of hematopoietic malignancies could be initiated. To meet that objective, production of ^211^At-BC8-B10 had to be translated from a chemistry laboratory to a cGMP suite. While the chemical steps were similar for both production locations, small changes in the chemistry were required to conduct the preparation in the cGMP suite due to the sterility requirements. Sterility requirements mandated that conducting the ^211^At labeling and purification of the labeled product be done in closed vessels, which resulted in changing how reagents were added or transferred. These changes, along with FDA requirements for documentation (batch records) resulted in extending the overall time it requires for production when done in a laboratory setting. While not discussed herein, training of personnel in aseptic techniques and working in a cGMP suite under a fair number of procedural SOPs was a significant challenge to initiating the translation. In the end, a cGMP production of ^211^At-BC8-B10 involving production of multiple components has been successfully developed. Quality control methods and release criteria for the clinical grade ^211^At-BC8-B10 have been established. Qualification production runs involving ^211^At-labeling have demonstrated high reproducibility in producing sterile, pure ^211^At-BC8-B10. Importantly, ^211^At-BC8-B10 has been produced in quantities that approach the maximum amount anticipated for patient treatment. Indeed, while other investigators have produced ^211^At-labeled antibodies for clinical trials [25], the quantities reported herein may be the highest produced to date. We have used the data presented herein to support a successful Investigational New Drug (IND) application to the United States Food and Drug Administration enabling a clinical trial using ^211^At-BC8-B10 in treatment of advanced hematopoietic malignancies (IND 1336100). That clinical trial has begun (NCT03128034).

## Supporting information

S1 Fig Chemical synthesis reactions used to prepare *p*-isothiocyanato-phenethyl-ureido-*closo*-decaborate(2-), the B10-NCS reagent. (PDF)

S2 Fig Chemical purity of the B10-NCS reagent analyzed by HPLC as determined by reversed-phase HPLC using UV detection (bottom chromatogram) and ELSD detection (top chromatogram).
(PDF)

S3 Fig Mass spectral data (parent peak isotope pattern) for the B10-NCS reagent. (PDF)

S4 Fig Manufactured cGMP grade BC8 MAb used in subsequent clinical production of BC8-B10. (PDF)

S5 Fig Size-exclusion HPLC chromatograms showing UV absorbing peaks for BC8 (top panel) and BC8-B10 (bottom panel).
(PDF)

S6 Fig FACS binding assessment of BC8-B10 using Jurkat cells (panel A) or Ramos cells (panel B) with primary antibodies at 5 μg/mL.
(PDF)

S7 Fig Stained IEF gel showing production product of BC8-B10 (lane 3) against a reference standard BC8-B10 (lane 6) and a reference standard BC8 (lane 8). (PDF)

S8 Fig IEF gel analysis of BC8 and BC8-B10 (left panel) showing bands from materials identified for lanes 1–10. (PDF)

S9 Fig Quantification of number of B10-NCS moieties per BC8 molecule using mass spectral analyses. (PDF)

S10 Fig Chromatograms of isolated ^211^At. Top chromatogram used gamma detector and bottom used UV detector.
(PDF)

S11 Fig Radio-ITLC scan of isolated ^211^At. Astatide moves to near solvent front (100%) on the iTLC plate.
(PDF)

S12 Fig Pictures showing apparatus used for filter integrity bubble test. (PDF)

S13 Fig SE-HPLC chromatograms of three standard solutions (panels A–C) containing BC8-B10 and a solution of 211At-BC8-B10 with an unknown amount of protein (panel D).
(PDF)

S1 Table Testing conducted and release criteria for each component in ^211^At-BC8-B10 production. (PDF)
