# Supplementary material for: cGMP production of astatine-211-labeled anti-CD45 antibodies for use in allogeneic hematopoietic cell transplantation for treatment of advanced hematopoietic malignancies
Source: PLoS One. 2018 Oct 18;13(10):e0205135. doi: 10.1371/journal.pone.0205135 (PMC6193629; doi:10.1371/journal.pone.0205135)
Supplement: S1 Fig — (PDF) [file pone.0205135.s001.pdf]

Supporting information for production of B10-NCS (Production Step 1)

Note: Synthesis of B10-NCS was conducted in the Department of Radiation Oncology's Molecular Radiotherapy Research Laboratory at the University of Washington. A Certificate of Analysis was provided for its use in the MAb conjugation step.

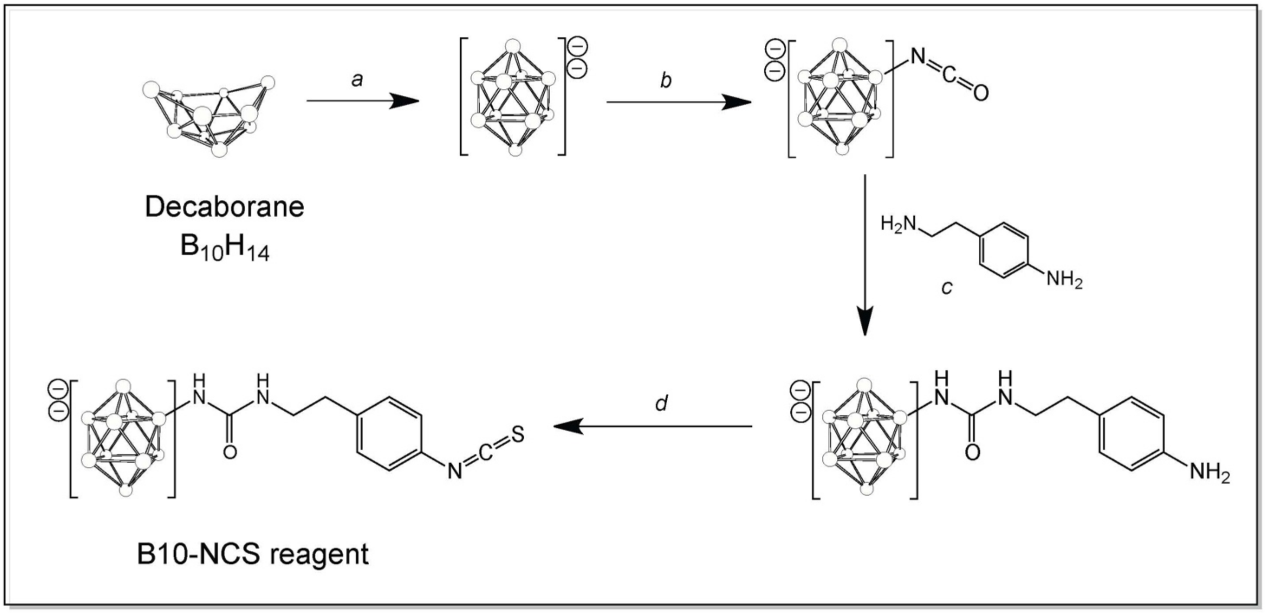

Figure S1. Chemical synthesis reactions used to prepare *p*-isothiocyanato-phenethyl-ureido-*closo*-decaborate(2-), the **B10-NCS** reagent. The starting decaborane ($B_{10}H_{14}$) is commercially available (Sigma-Aldrich Corp., St. Louis, MO). Boron cage structures show circles for boron atoms, protons are not shown for simplicity. Cationic counterions for borate(2-) structures are triethylammonium ions. Scale-up of reaction conditions used to obtain B10-NCS for clinical preparations is described below. ^{1}H and ^{11}B NMR was the same as those previously reported in ref # 13. Mass spectral data used to confirm identity of each product is provided.

Reaction conditions used for production scale of B10-NCS

a. $B_{10}H_{14}$ (5 g, 40.9 mmol) was dissolved in xylenes (80 mL) in a 250 mL three-necked flask equipped with a thermometer, a reflux condenser, and an oil bath. The flask was flushed with argon and then NEt_3 (108 mmol, 15 mL) was added dropwise over 5 min. The temperature of the solution was raised to about 105 °C and the reaction mixture was stirred for 3 h under argon. During this stage hydrogen gas was evolved. The temperature was then raised until the xylenes refluxed (about 140 °C), and the solution was stirred at the reflux temperature for 5 h. The solution was cooled to room temperature, then EtOAc (150 mL) was added, followed by stirring at room temperature for 1 h, then filtration. A pale-yellow solid was obtained, which was washed with EtOAc (3 x 40 mL) to remove the most of the yellow color and impurities. The compound was dried under open air for 2 h. The crude product was dissolved in acetonitrile (10 mL), then EtOAc (120 mL) was added to the solution slowly at room temperature with stirring. The white precipitate was filtered, washed with EtOAc (3 x 40 mL), dried under open air for 2 h, then dried under vacuum for 16 h. Yield 12.87 g (98%). mp 232-233 °C. HRMS (ES) $B_{10}H_{10}$ (M-) Calcd: 120.1713, Found: 120.1791.

b. Oxalyl chloride (1.31 mL, 15.5 mmol) was added dropwise to a solution of $[Et_3NH]_2$ $B_{10}H_{10}$ (5.0 g, 15.5 mmol) and anhydrous CH_3CN (150 mL) at ice bath temperature. The resultant solution was stirred at ice bath temperature for 0.5 h. After ice bath was removed, the solution was stirred for another 0.5 h. NaN_3 (2.12 g, 32.5 mmol) was added, and the solution was allowed to stir at room temperature for 16 h. The reaction mixture was filtered, evaporated to dryness. The crude product was purified via preparative chromatography on a Biotage instrument using a C18 FLASH 25+M column. The column was eluted using a gradient mixture composed of MeOH and 0.05 M triethylammonium acetate, starting with 100% 0.05 M triethylammonium acetate, then increasing to 100% MeOH over the next 15 min. Yield 3.74 g (66.4%). HRMS (ES) $CH_9B_{10}NO$ (M-) Calcd: 161.1615, Found 161.1724.

c. A solution of $[Et_3NH]_2$ $B_{10}H_9$-N=C=O (1.25 g, 3.44 mmol), *p*-NH_2-Ph-$CH_2CH_2NH_2$ (0.609 g, 4.47 mmol), NEt_3 (0.719 mL, 5.16 mmol), and anhydrous DMF (15 mL) was stirred and heated by microwave at 80 °C for 3 h. The crude product was purified via preparative chromatography on a Biotage instrument using a C18 FLASH 25+M column. The column was eluted using a gradient mixture composed of MeOH and 0.05 M triethylammonium acetate, starting with 100% 0.05 M triethylammonium acetate, then increasing to 100% MeOH over the next 15 min. Yield 1.22 g (71.0%). HRMS (ES) $C_9H_{21}B_{10}N_3O$ (M-) Calcd: 297.2615, found: 297.2710

d. A solution containing $[Et_3NH]_2$ $B_{10}H_9$-NHCONH-CH_2CH_2-Ph-NH_2 (10 mg, 0.02 mmol), 1,1'-thiocarbonyldiimidazole (5.15 mg, 0.026 mmol) and anhydrous DMF (0.4 mL) was stirred at room temperature for 1 h. The reaction mixture was washed 3 times with 15 mL of 20% EtOAc/hexanes, then the remained light-yellow product was dried under vacuum for 1 h. Yield 10.8 mg (100%). HRMS (ES) $C_{10}H_{19}B_{10}N_3OS$ (M-) Calcd: 339.2179, Found: 339.2180.
