# Supplementary material for: cGMP production of astatine-211-labeled anti-CD45 antibodies for use in allogeneic hematopoietic cell transplantation for treatment of advanced hematopoietic malignancies
Source: PLoS One. 2018 Oct 18;13(10):e0205135. doi: 10.1371/journal.pone.0205135 (PMC6193629; doi:10.1371/journal.pone.0205135)
Supplement: S2 Fig — Chemical purity of the B10-NCS reagent analyzed by HPLC as determined by reversed-phase HPLC using UV detection (bottom chromatogram) and ELSD detection (top chromatogram). (PDF) [file pone.0205135.s002.pdf]

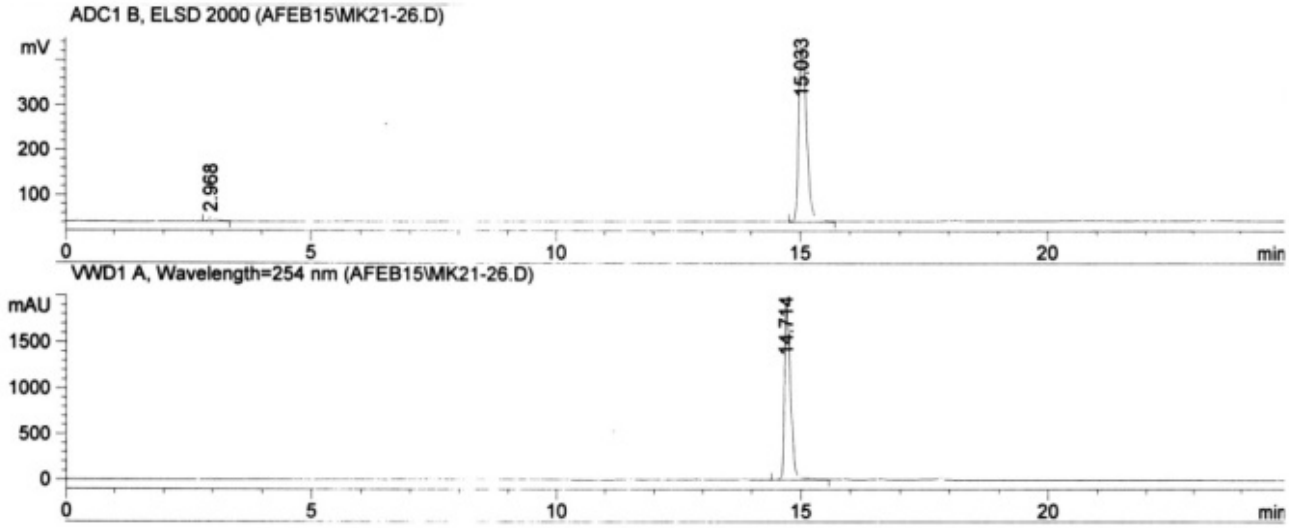

Figure S2. Chemical purity of the B10-NCS reagent analyzed by HPLC as determined by reversed-phase HPLC using UV detection (bottom chromatogram) and ELSD detection (top chromatogram). The ELSD detector is later in the effluent flow, as noted by the later retention time. The RP-HPLC system consisted of a Hewlett-Packard quaternary 1050 gradient pump, a variable wavelength UV detector (254 nm), and an Alltech ELSD 2000 evaporative light-scattering detector (Deerfield, IL). Hewlett-Packard HPLC ChemStation software was used for the analyses of HPLC data.
