# Supplementary material for: cGMP production of astatine-211-labeled anti-CD45 antibodies for use in allogeneic hematopoietic cell transplantation for treatment of advanced hematopoietic malignancies
Source: PLoS One. 2018 Oct 18;13(10):e0205135. doi: 10.1371/journal.pone.0205135 (PMC6193629; doi:10.1371/journal.pone.0205135)
Supplement: S3 Fig — (PDF) [file pone.0205135.s003.pdf]

A: Measured

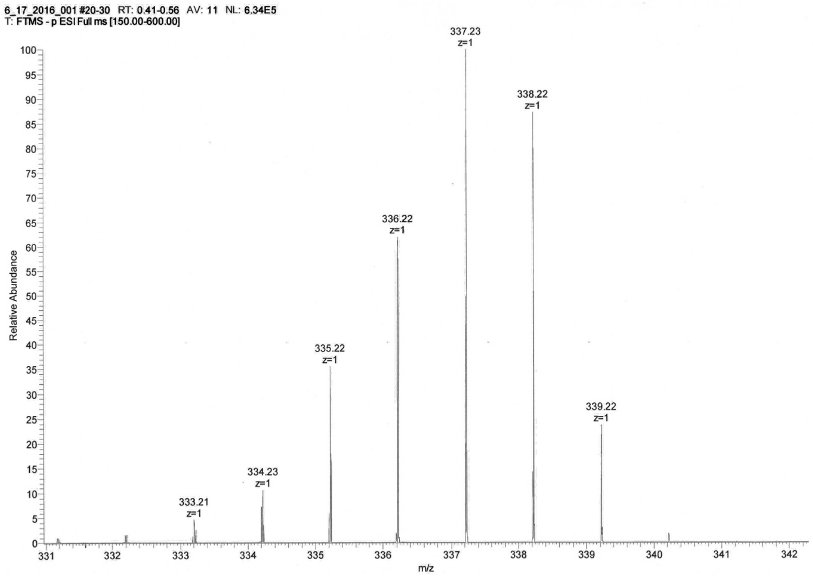

B: Calculated

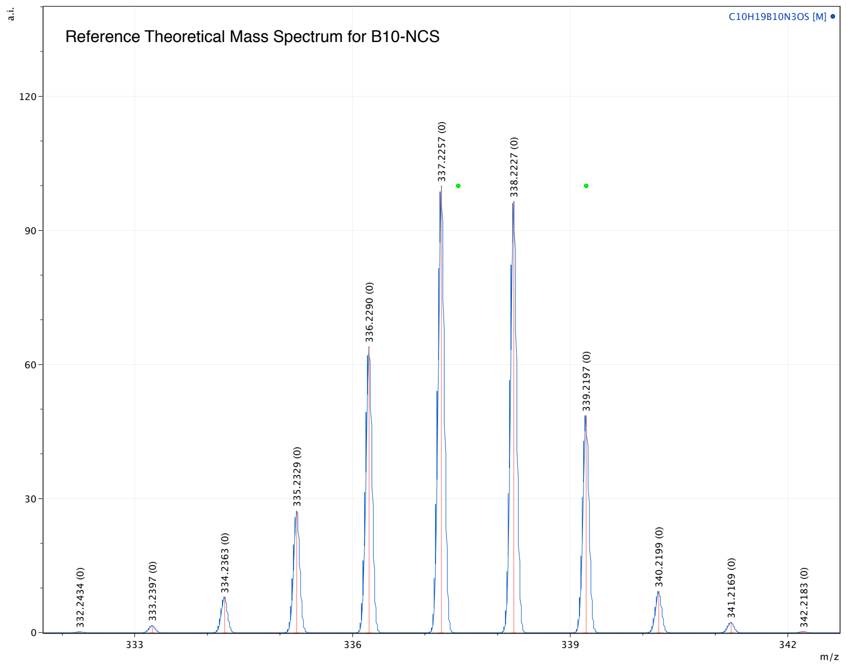

Figure S3. Mass spectral data (parent peak isotope pattern) for the B10-NCS reagent. Top partial spectrum is that of the isolated B10-NCS and bottom partial spectrum has been calculated. Note that isotopic abundance pattern is very similar to that calculated. Matching exact mass can be done with any of the major masses.
