# Supplementary material for: cGMP production of astatine-211-labeled anti-CD45 antibodies for use in allogeneic hematopoietic cell transplantation for treatment of advanced hematopoietic malignancies
Source: PLoS One. 2018 Oct 18;13(10):e0205135. doi: 10.1371/journal.pone.0205135 (PMC6193629; doi:10.1371/journal.pone.0205135)
Supplement: S4 Fig — (PDF) [file pone.0205135.s004.pdf]

Supporting information for production of BC8 (Production Step 2)

Note: Production of BC8 was conducted in Biological Production Facility at the Fred Hutchinson Cancer Research Center under cGMP conditions

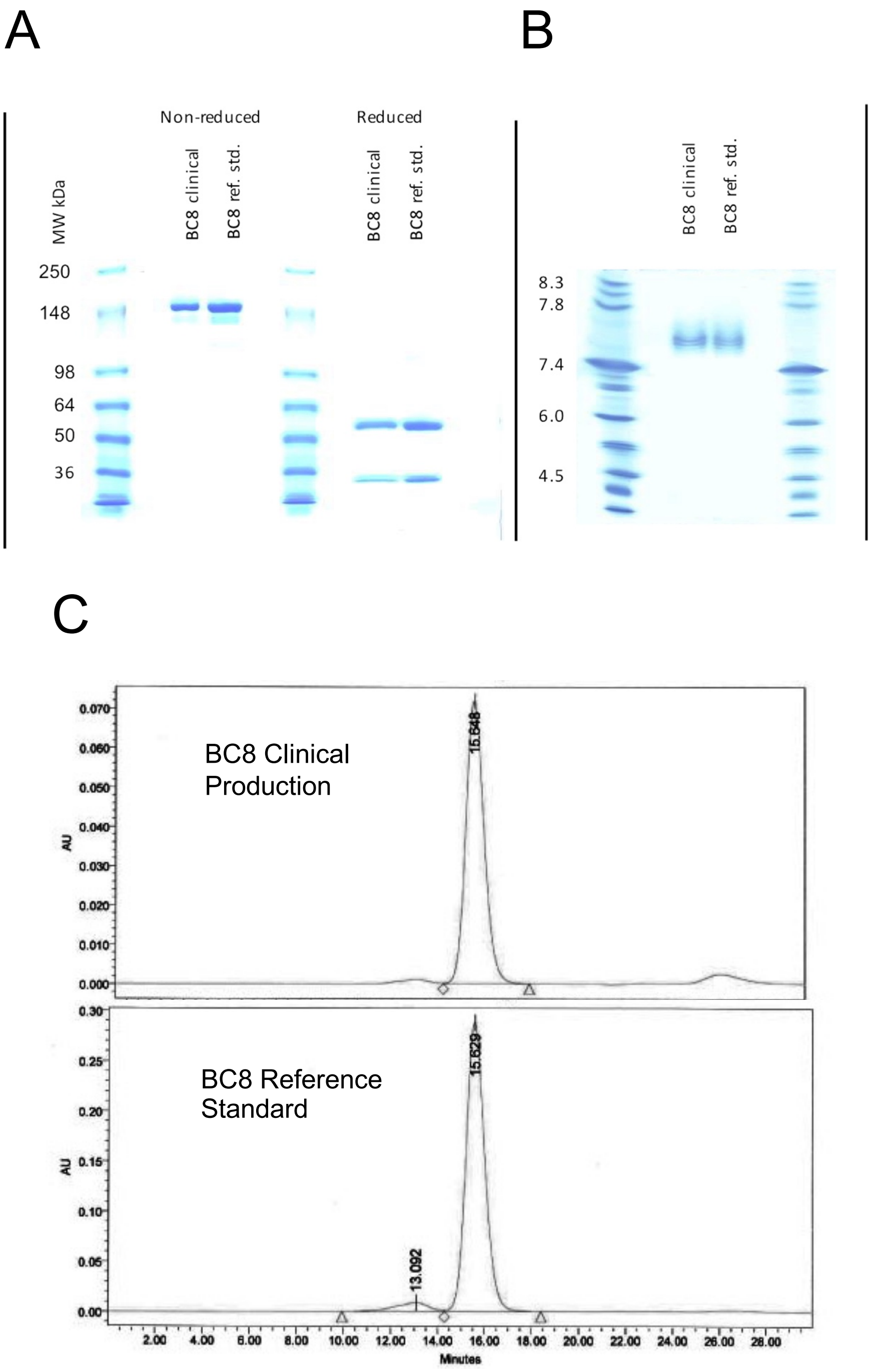

Figure S4. Manufactured cGMP grade BC8 MAb used in subsequent clinical production of BC8-B10. Analysis by SDS-PAGE (panel **A**), IEF (panel **B**) and SE-HPLC (panel **C**) demonstrate high purity, identity and monomeric status. The data from these assays, in conjunction with additional release criteria, signified that the BC8 was in compliance with regulatory standards.
