# Supplementary material for: cGMP production of astatine-211-labeled anti-CD45 antibodies for use in allogeneic hematopoietic cell transplantation for treatment of advanced hematopoietic malignancies
Source: PLoS One. 2018 Oct 18;13(10):e0205135. doi: 10.1371/journal.pone.0205135 (PMC6193629; doi:10.1371/journal.pone.0205135)
Supplement: S5 Fig — Size-exclusion HPLC chromatograms showing UV absorbing peaks for BC8 (top panel) and BC8-B10 (bottom panel). (PDF) [file pone.0205135.s005.pdf]

Supporting information for production of BC8-B10 (Production Step 3)

Note: Production of BC8-B10 was conducted in Biological Production Facility at the Fred Hutchinson Cancer Research Center under cGMP conditions

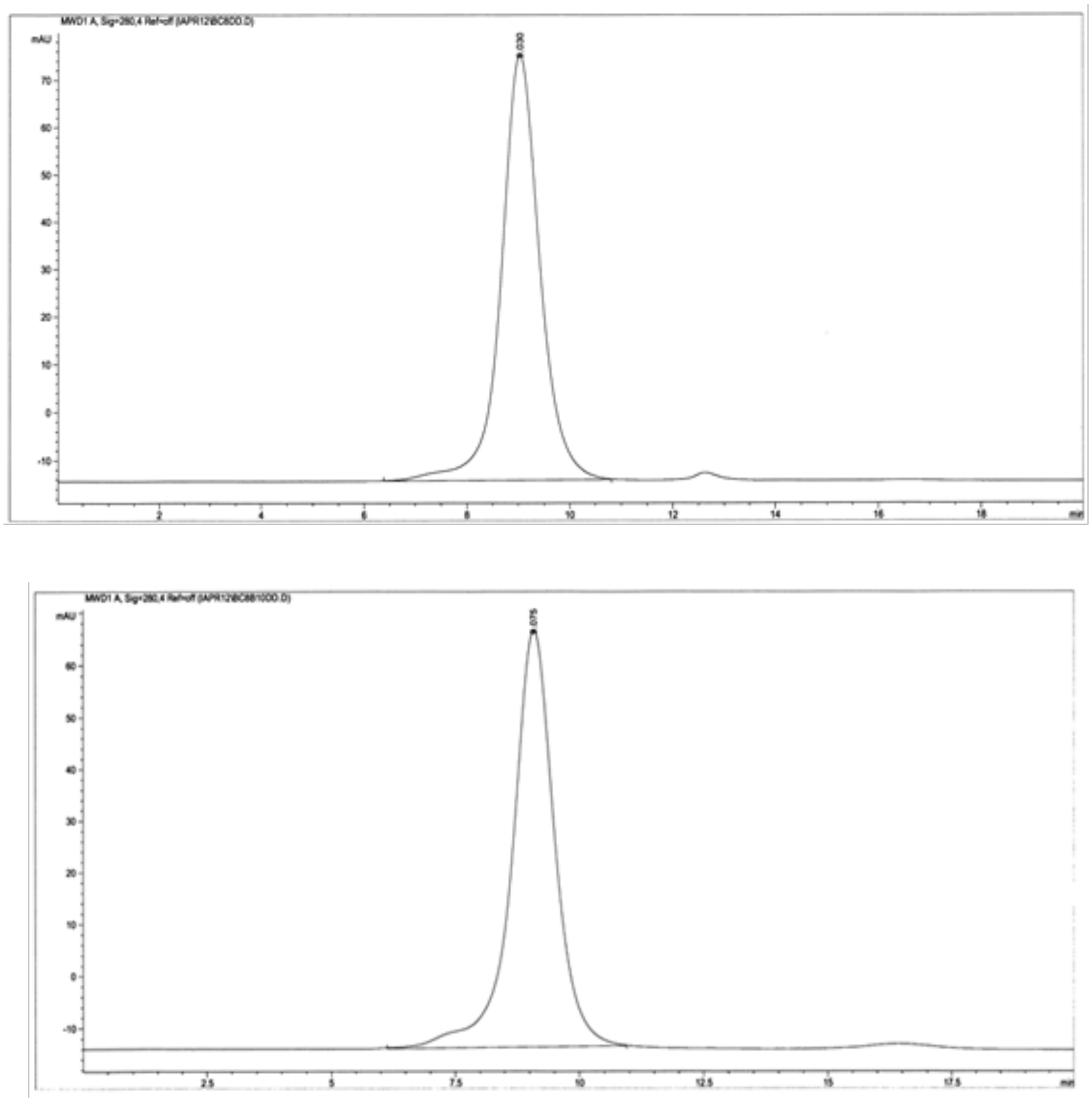

Figure S5: Size-exclusion HPLC chromatograms showing UV absorbing peaks for BC8 (top panel) and BC8-B10 (bottom panel). Analytical SE-HPLC was performed on a system consisting of Waters 2487 dual lambda detector asbsorbance 280 nm, Waters Delta 600 E pump and controller (integration calculated by Waters Empower Pro software) and a Protein Pak stainless steel 300SW column (7.5 x 300 mm, 10um; Water Corp., Milford, MA). The column was eluted using PBS at a flow rate of 1 mL/min.
