# Supplementary material for: cGMP production of astatine-211-labeled anti-CD45 antibodies for use in allogeneic hematopoietic cell transplantation for treatment of advanced hematopoietic malignancies
Source: PLoS One. 2018 Oct 18;13(10):e0205135. doi: 10.1371/journal.pone.0205135 (PMC6193629; doi:10.1371/journal.pone.0205135)
Supplement: S6 Fig — FACS binding assessment of BC8-B10 using Jurkat cells (panel A) or Ramos cells (panel B) with primary antibodies at 5 μg/mL. (PDF) [file pone.0205135.s006.pdf]

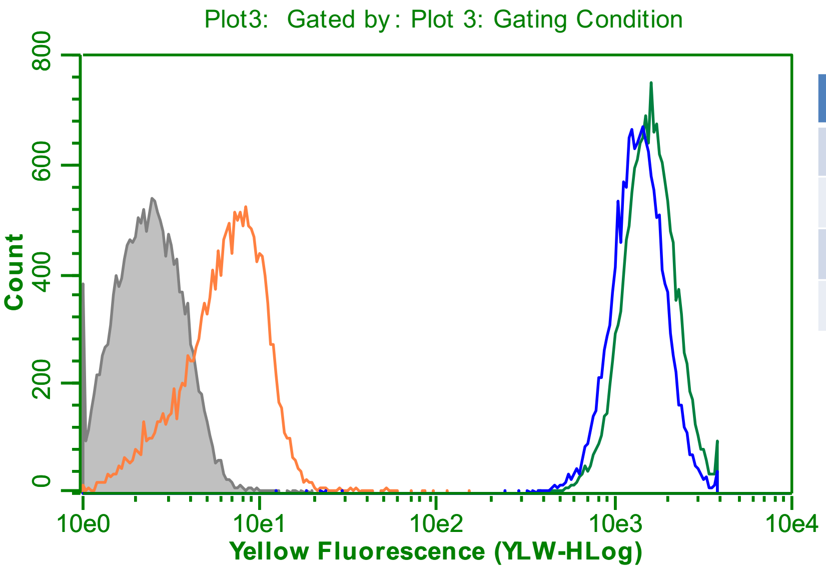

Test agent	MFI
Cells alone	2.64
BHV1	5.89
BC8 ctrl	1670
BC8.B10	1450

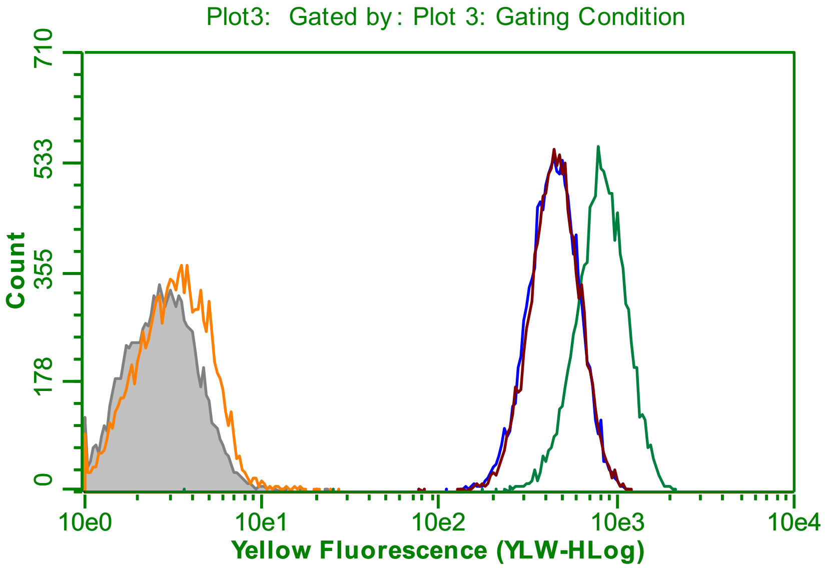

Test agent	MFI
Cells alone	3.05
BHV1	3.51
BC8 ctrl	858
BC8-B10 (w/oAc)	463
BC8-B10 (w/out oAc)	471

Figure S6. FACS binding assessment of BC8-B10 using Jurkat cells (panel A) or Ramos cells (panel B) with primary antibodies at 5 μg/mL. Secondary antibody used is anti-murine Fcγ-RPE. Colored histograms are as follows: shaded grey is cells alone, BHV1 control is orange, BC8 control is green, and test BC8-B10 is blue.
