# Supplementary material for: cGMP production of astatine-211-labeled anti-CD45 antibodies for use in allogeneic hematopoietic cell transplantation for treatment of advanced hematopoietic malignancies
Source: PLoS One. 2018 Oct 18;13(10):e0205135. doi: 10.1371/journal.pone.0205135 (PMC6193629; doi:10.1371/journal.pone.0205135)
Supplement: S7 Fig — (PDF) [file pone.0205135.s007.pdf]

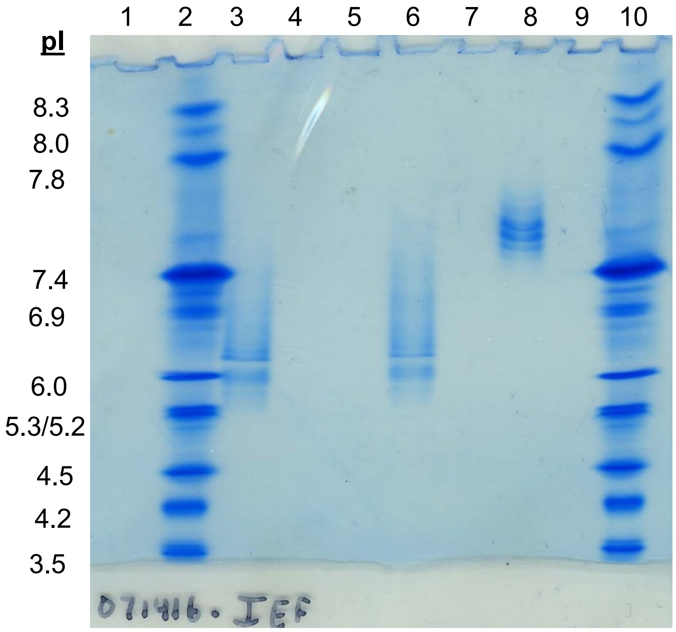

Lane 1: Blank
Lane 2 Serva IEF Marker pH 3-10
Lane 3: BC8-B10 Final Bulk 6/17/16, 5.1 mg/mL, 5 µg loaded
Lane 4: Blank
Lane 5: Blank
Lane 6: BC8-B10 in PBS, LN: 040616, 5.0 mg/mL, 5 µg loaded
Lane 7 Blank
Lane 8: BC8 Reference Standard, 5.0 mg/mL, 5 µg loaded
Lane 9: Blank
Lane 10: Serva IEF Marker pH 3-10

Figure S7. Stained IEF gel showing production product of BC8-B10 (lane 3) against a reference standard BC8-B10 (lane 6) and a reference standard BC8 (lane 8). IEF gel was an Invitrogen (Novex) precast gel, pH 3−10 (1.0 mm thick × 12 wells) run using a Novex PowerEase 500 instrument with the XCell II chamber, using the standard IEF program.
