# Supplementary material for: cGMP production of astatine-211-labeled anti-CD45 antibodies for use in allogeneic hematopoietic cell transplantation for treatment of advanced hematopoietic malignancies
Source: PLoS One. 2018 Oct 18;13(10):e0205135. doi: 10.1371/journal.pone.0205135 (PMC6193629; doi:10.1371/journal.pone.0205135)
Supplement: S8 Fig — (PDF) [file pone.0205135.s008.pdf]

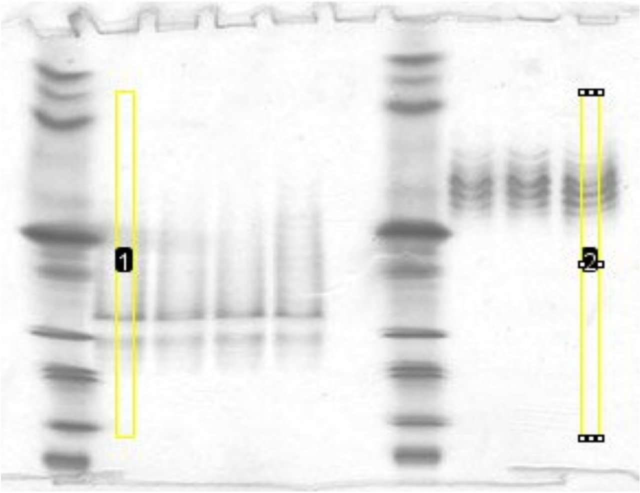

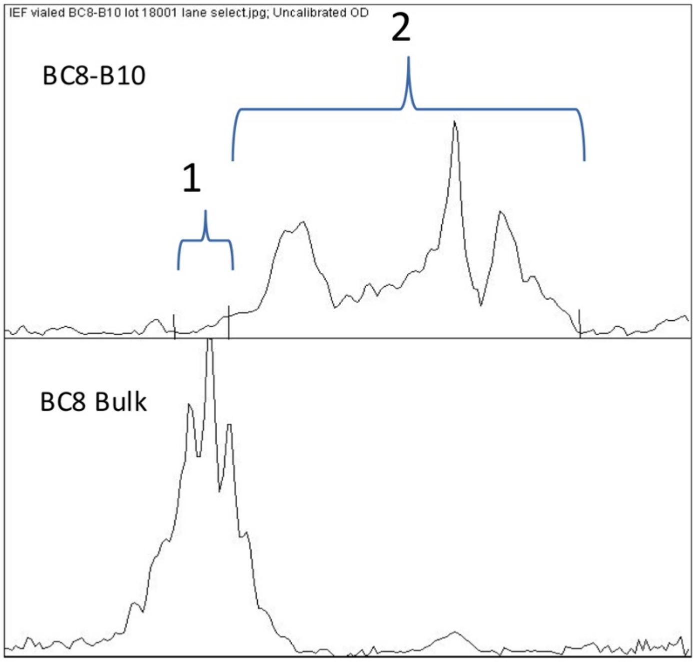

Lane 1: Serva IEF Marker pH 3-10
Lane 2 BC8-B10 Vialed Product, Lot 18001
Lane 3: BC8-B10 Reference STD, Lot#18001, vial #099,
Lane 4: BC8-B10 Reference STD, Lot#18001, vial #091,
Lane 5: BC8-B10 in PBS, pilot LN 040616
Lane 6: Blank
Lane 7 Serva IEF Marker pH 3-10
Lane 8: BC8 Reference Standard lot 2015,
Lane 9: BC8 Final Bulk 2016, 8/1/16,
Lane 10: BC8 DEAE Pool 2000 (W,X)

Calculation of % BC8 in BC8-B10
Areas under blue lines
Area 1 554
Area 2 22625

(Area 1/Areas 1+2)*100 =
< 2.4% unmodified BC8

Figure S8. IEF gel analysis of BC8 and BC8-B10 (left panel) showing bands from materials identified for lanes 1 – 10. The yellow overlay shows the bands analyzed to evaluate the percent unmodified BC8 in the BC8-B10 conjugate. Right panel shows gel band density graphs (right panel) using the NIH ImageJ program. Area 1 designation in top density graph shows area where one would expect peaks from unaltered BC8 if it was present. Area 2 in the top density graph shows area where new materials [e.g. BC8-B10, BC8-$(B10)_2$, BC8-$(B10)_3$, etc.] are found. Background density was corrected using ImageJ ROI background subtraction algorithm. IEF gel was an Invitrogen (Novex) precast gel, pH 3−10 (1.0 mm thick × 12 wells) run using a Novex PowerEase 500 instrument with the XCell II chamber, using the standard IEF program.
