# Supplementary material for: cGMP production of astatine-211-labeled anti-CD45 antibodies for use in allogeneic hematopoietic cell transplantation for treatment of advanced hematopoietic malignancies
Source: PLoS One. 2018 Oct 18;13(10):e0205135. doi: 10.1371/journal.pone.0205135 (PMC6193629; doi:10.1371/journal.pone.0205135)
Supplement: S9 Fig — (PDF) [file pone.0205135.s009.pdf]

Parent Peak from MS of BC8 sample 1

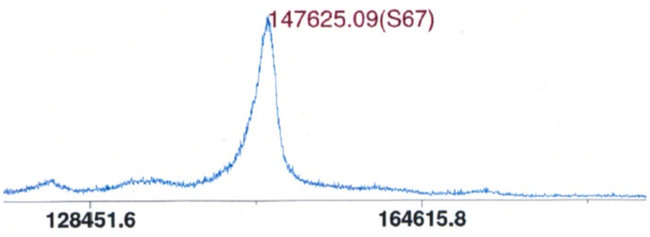

Parent Peak from MS of BC8 sample 2

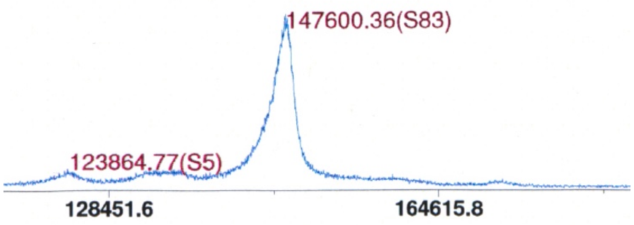

Parent Peak from MS of BC8-B10 sample 1

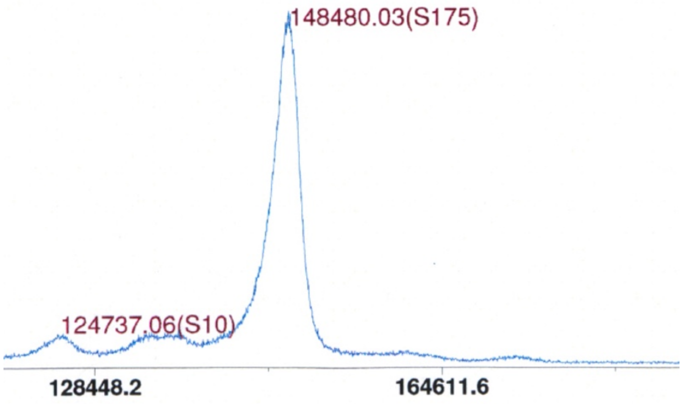

Parent Peak from MS of BC8-B10 sample 2

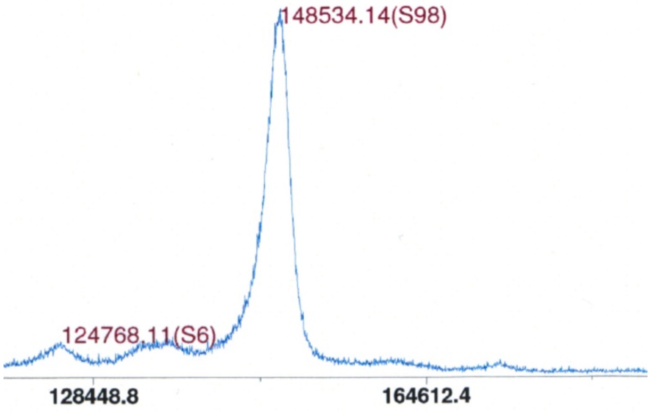

Figure S9. Quantification of number of B10-NCS moieties per BC8 molecule using mass spectral analyses. Shown are parent peaks from mass spectra of desalted BC8 and BC8-B10 samples. Desalting was done using ZipTip pipette tips (Millipore Sigma, St. Louis, MO). An average mass was obtained for BC8 (147,613, Da) and BC8-B10 (148,507 Da) from the two runs. The difference in average mass (894 Da) was divided by the mass of B10-NCS (337 Da) to estimate the number of conjugates per MAb molecule, i.e. ~2.7 conjugates/BC8 molecule. Mass spectra were obtained at Fred Hutch Applied Biosciences SciEx 4800 Maldi TOF/TOF Analyzer (Wellborn TX) using linear mode with positive polarity.
