# Supplementary material for: cGMP production of astatine-211-labeled anti-CD45 antibodies for use in allogeneic hematopoietic cell transplantation for treatment of advanced hematopoietic malignancies
Source: PLoS One. 2018 Oct 18;13(10):e0205135. doi: 10.1371/journal.pone.0205135 (PMC6193629; doi:10.1371/journal.pone.0205135)
Supplement: S10 Fig — Top chromatogram used gamma detector and bottom used UV detector. (PDF) [file pone.0205135.s010.pdf]

Supporting Information for production of Na[^{211}At]At (Production Step 4)

Note: Production of Na[^{211}At]At was conducted in Department of Radiation Oncology's Radionuclide Production Laboratory at the University of Washington. A Certificate of Analysis was provided for its use in the ^{211}At-labeling step.

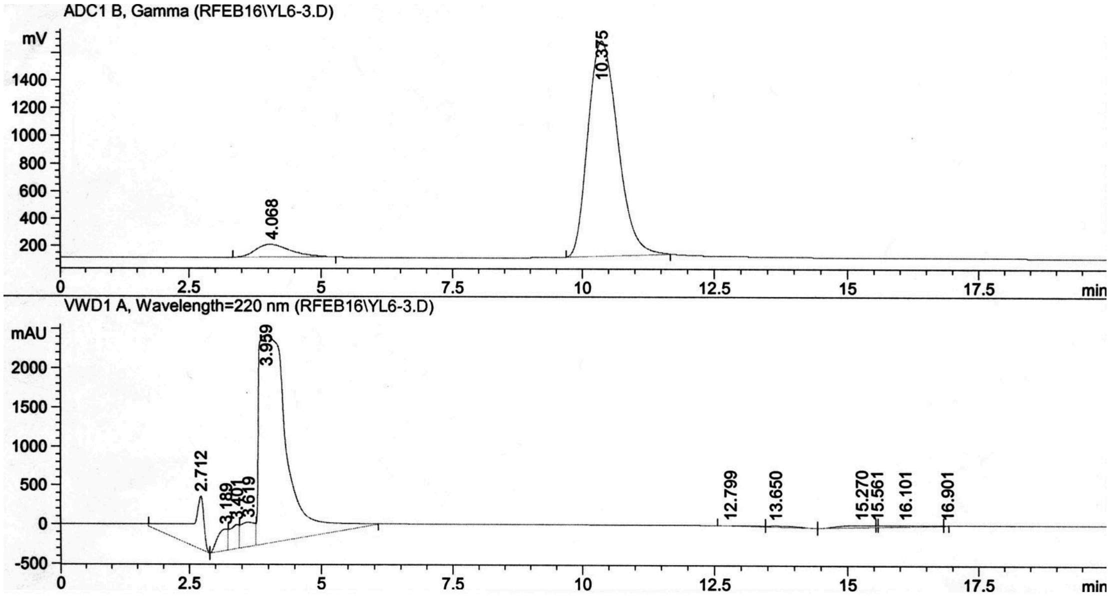

Figure S10. Chromatograms of isolated ^{211}At. Top chromatogram used gamma detector and bottom used UV detector. Gamma peak at 10.4 min is astatide. Radio-HPLC was conducted on a Hewlett-Packard model 1050 HPLC (Hewlett-Packard Company, Palo Alto, CA) with a Beckman Model 170 Radioisotope Detector (Beckman-Coulter, Brea, CA). The ^{211}At-containing solution was analyzed on a Dionex IonPac AS-20 anion exchange column with a Dionex AG-20 guard column (Dionex, Sunnyvale, CA), eluting with a 50 mM NaOH solution at a flow rate of 1.3 mL/min in isocratic mode.
