# Supplementary material for: cGMP production of astatine-211-labeled anti-CD45 antibodies for use in allogeneic hematopoietic cell transplantation for treatment of advanced hematopoietic malignancies
Source: PLoS One. 2018 Oct 18;13(10):e0205135. doi: 10.1371/journal.pone.0205135 (PMC6193629; doi:10.1371/journal.pone.0205135)
Supplement: S11 Fig — Astatide moves to near solvent front (100%) on the iTLC plate. (PDF) [file pone.0205135.s011.pdf]

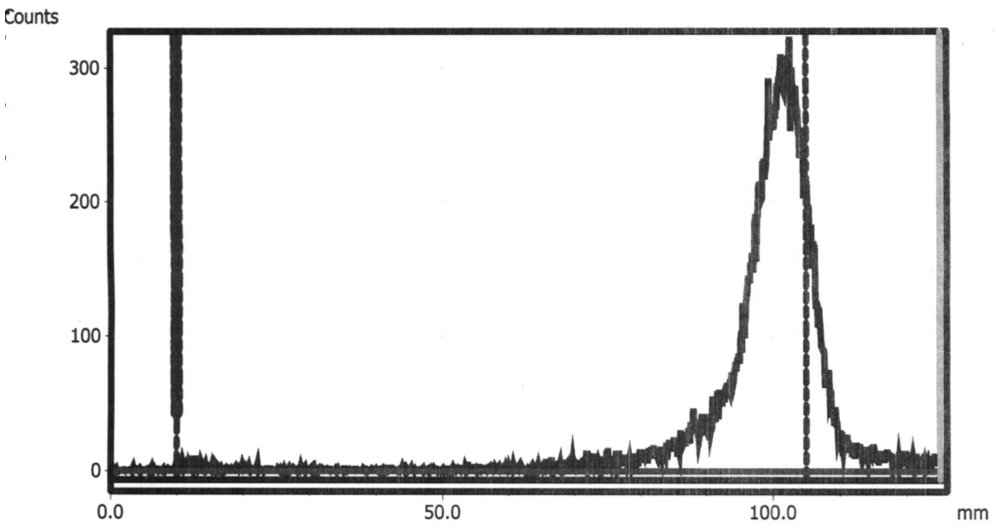

Figure S11. Radio-ITLC scan of isolated ^{211}At. Astatide moves to near solvent front (100%) on the iTLC plate. The iTLC analyses were conducted using a LabLogic ScanRAM scanner equipped with a 1-mm NaI probe and the Laura software. Radio-iTLC analyses were carried out on iTLC-SG chromatographic strips (Agilent, Santa Clara, CA) eluted using 3 M NH_4OH saturated *n*-butanol.
