# Supplementary material for: cGMP production of astatine-211-labeled anti-CD45 antibodies for use in allogeneic hematopoietic cell transplantation for treatment of advanced hematopoietic malignancies
Source: PLoS One. 2018 Oct 18;13(10):e0205135. doi: 10.1371/journal.pone.0205135 (PMC6193629; doi:10.1371/journal.pone.0205135)
Supplement: S12 Fig — (PDF) [file pone.0205135.s012.pdf]

## Supporting Information for production of $^{211}$At-BC8-B10 (Production Step 5)

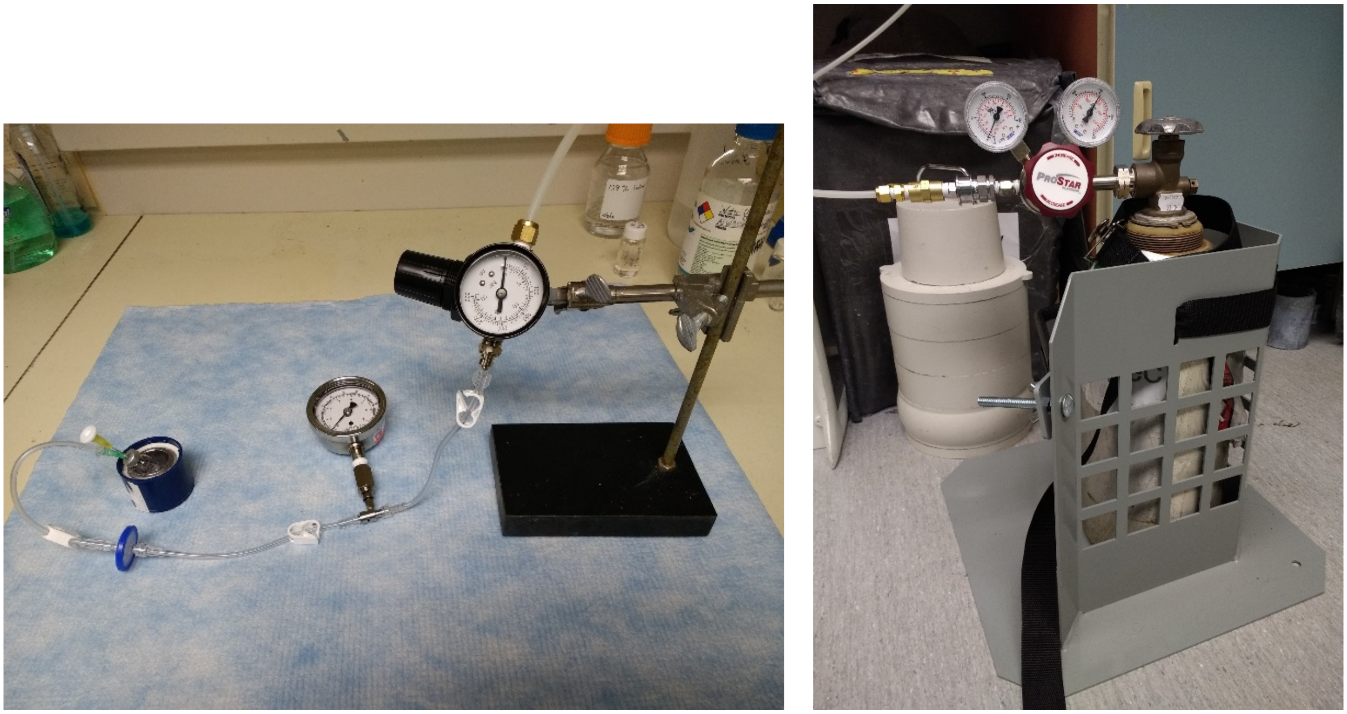

**Figure S12.** Pictures showing apparatus used for filter integrity bubble test. Right panel shows air tank with 2-stage regulator for pressurizing the setup. Left panel shows air line from tank to (in order) (a) step-down pressure regulator, (b) bubble point/filter integrity tester (Laboratory Sales and Services, LLC, Branchburg, NJ), (c) 0.1 μm syringe filter used in production of $^{211}$At-BC8-B10, (d) line/needle leading to water in vial with HEPA filter outlet.
