# Supplementary material for: cGMP production of astatine-211-labeled anti-CD45 antibodies for use in allogeneic hematopoietic cell transplantation for treatment of advanced hematopoietic malignancies
Source: PLoS One. 2018 Oct 18;13(10):e0205135. doi: 10.1371/journal.pone.0205135 (PMC6193629; doi:10.1371/journal.pone.0205135)
Supplement: S13 Fig — SE-HPLC chromatograms of three standard solutions (panels A–C) containing BC8-B10 and a solution of 211At-BC8-B10 with an unknown amount of protein (panel D). (PDF) [file pone.0205135.s013.pdf]

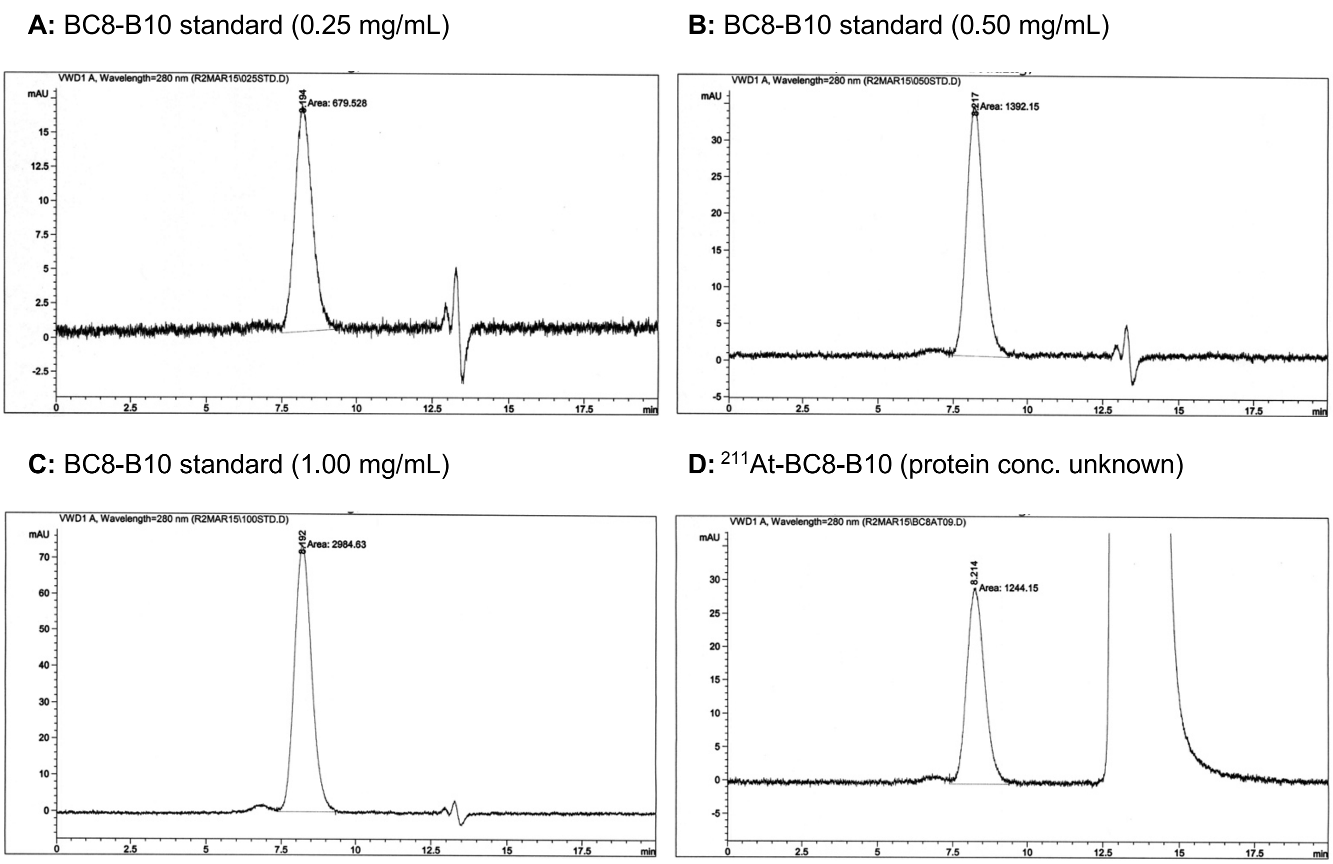

Figure S13. SE-HPLC chromatograms of three standard solutions (panels A – C) containing BC8-B10 and a solution of ^{211}At-BC8-B10 with an unknown amount of protein (panel D). A least squares analysis was run on the peak areas for the standards to get a standard curve (r^2 = 0.9993). The large peak at 12.5-15 min in panel D is from ascorbic acid. Using the area under the protein peak on panel D (1244) a protein concentration of 0.43 mg/mL was obtained.
