# Supplementary material for: cGMP production of astatine-211-labeled anti-CD45 antibodies for use in allogeneic hematopoietic cell transplantation for treatment of advanced hematopoietic malignancies
Source: PLoS One. 2018 Oct 18;13(10):e0205135. doi: 10.1371/journal.pone.0205135 (PMC6193629; doi:10.1371/journal.pone.0205135)
Supplement: S1 Table — (PDF) [file pone.0205135.s014.pdf]

Table S1: Testing Conducted and Release Criteria for Each Component in ^{211}At-BC8-B10 Production

Table lists the type of test conducted, where it was conducted the release criteria that was used and examples of results obtained for one of the lots produced.

Bulk Purified BC8 MAb (GMP-Used as intermediate)

Test	Lab*	Release Specification	Results Obtained
Sterility	FH-BPF	Negative	Negative
Endotoxin	FH-BPF	< 5 EU/mg MAb	<0.04 EU/mg (<0.5 EU/mL)
Isotype	FH-BPF	IgG_1	IgG_1 identified
SDS-PAGE	FH-BPF	Identification of intact MAb (non-reduced); Heavy/Light chains when reduced	Conforms to standard
IEF	FH-BPF	Confirm expected isoforms (bands from pI 7.1-7.9)	Conforms to standard
Protein A	FH-BPF	< 3 ppm	0.32 ppm
SE-HPLC	FH-BPF	Confirm monomeric status	Monomeric species, 100%
UV; A280 Protein	FH-BPF	> 0.1 mg/mL	1.28 mg/mL
Residual Bovine IgG	FH-BPF	Report	0.08 ppm
Cell Binding By FACS	FH-TL	> 70% to reference standard	111% to ref. std.

B10-NCS Conjugation Reagent (non-GMP-Used as Raw Material with Certificate of Analysis)

Test	Lab*	Release Specification	Results Obtained
RP-HPLC	UW-MRRL	Retention time conforms to reference standard (12.8 min)	Conforms to standard
RP-HPLC, ELSD	UW-MRRL	≥ 90% (area %) in peak	100% in peak
Mass Spectra	UW-MRRL	Mass conforms to theoretical mass (without counterions) 337.23 Da (100%) – has B10 isotope pattern	337.23 Da (100%)

Purified Bulk BC8-B10 MAb Conjugate (cGMP-Used as intermediate)

Test	Lab*	Release Specification	Results Obtained
Sterility	FH-BPF	Negative	Negative
Endotoxin	FH-BPF	< 10 EU/mg BC8-B10	0.1 EU/mg
SDS-PAGE	FH-BPF	>90% ~150 kDa band non-reduced; >90% H&L chains reduced	Passed
IEF	FH-BPF	Confirm expected isoforms	Passed
SE-HPLC	FH-BPF	Confirm monomeric status (>90%)	Monomeric, 96.4%

Vialed BC8-B10 MAb Conjugate (cGMP-Used as intermediate)

Test	Lab*	Release Specification	Results Obtained
MALDI-TOF MS	FH-BPF	Report mass spectra data	Found avg. 2.7 molar equivalents
Immunoreactivity	FH-TL	≥ 70% similar to labeled control	Ramos: 87%, Raji:93%
General Safety	FH-TL	CFR 21. 610.11	Met requirements
Sterility	FH-BPF	Tests at 3, 7 & 14 days negative	Negative
Endotoxin	FH-BPF	≤ 10 EU/mg BC8-B10	< 1 EU/mg
SDS-PAGE	FH-BPF	> 90% ~150 kDa band, non-reducing > 90% heavy & light chains, reducing	Passed
UV; A280 Protein	FH-BPF	4.5-5.5 mg/mL (predefined)	5.1 mg/mL

[^{211}At]NaAt (non-GMP-Used as Raw Material with Certificate of Analysis)

Test	Lab*	Release Specification	Results Obtained
Radio-TLC (Identity)	UW-RPL	Conforms to ^{125}I-Labeled Reference Standard	Passed
Radio-TLC (Chromat. Purity)	UW-RPL	≥ 85% (area %) Na[^{211}At]At	95.9%
HPGe (Radionuclidic Purity)	UW-RPL	>99+% ^{211}At (no ^{210}At) - test quarterly	Passed

^{211}At-BC8-B10 (cGMP-Final Product for Administration-Batch Record)

Test	Lab*	Release Specification	Results Obtained
Quantity of Radiolabeled Product	UW-NMRL	Set by weight of patient and dose prescribed (μCi/kg) ± 20 at midpoint of injection	Dose +10% delivered midpoint
Sterility	UW-NMRL	Tests at 3, 7 & 14 days negative Conducted, but not used for release due to half-life	Negative - post administration
Pyrogenicity	UW-NMRL	< 5.0 EU/kg/hr	Passed
iTLC purity	UW-NMRL	≥ 90%	97.2%
FACS cell binding	UW-NMRL	>50% at time of assay; Conducted, but not used for release due to half-life	Passed

*Laboratories are: FH-BPF, Fred Hutch Biological Production Facility; FH-TL, Fred Hutch Testing Laboratory; UW-MRRL, University of Washington Molecular Radiotherapy Research Laboratory; UW-RPL, University of Washington Radionuclide Production Laboratory; UW-NMRL, University of Washington Nuclear Medicine Radiolabeling Laboratory
